# Factors influencing oral health behaviours, access and delivery of dental care for autistic children and adolescents: A mixed‐methods systematic review

**DOI:** 10.1111/hex.13544

**Published:** 2022-06-18

**Authors:** Jo Erwin, Martha Paisi, Sarah Neill, Lorna Burns, Isaac Vassallo, Abigail Nelder, Jemma Facenfield, Urshla Devalia, Tara Vassallo, Robert Witton

**Affiliations:** ^1^ Peninsula Dental School University of Plymouth Plymouth UK; ^2^ School of Nursing and Midwifery University of Plymouth Plymouth UK; ^3^ Peninsula Dental Social Enterprise Plymouth UK; ^4^ School of Engineering, Computing and Mathematics University of Plymouth Plymouth UK; ^5^ Royal National ENT and Eastman Dental Hospital University College London Hospitals NHS Foundation Trust London UK; ^6^ Plymouth Institute of Education University of Plymouth Plymouth UK; ^7^ National Autistic Society—Plymouth & District Branch Plymouth UK

**Keywords:** access to dental care, autism, delivery of dental care, dental care, narrative systematic review, oral health

## Abstract

**Background:**

Autistic children and young people (CYP) experience oral health (OH) inequalities. They are at high risk of dental disease and show significant levels of unmet need in relation to OH and access to dental care.

**Aim:**

This study aimed to gather evidence on the factors that influence OH behaviours, access to and delivery of dental care for autistic CYP.

**Design:**

This was a mixed‐methods narrative systematic review.

**Data Sources:**

Embase, Web of Science, Dentistry & Oral Sciences Source, MEDLINE, Psychinfo, Scopus, CINAHL, SocINDEX and grey literature were the data sources for this study.

**Review Methods:**

A systematic search was conducted for qualitative, quantitative and mixed‐methods research studies from countries with a High Development Index that related to OH behaviours, access to and delivery of dental care for autistic CYP. Results were analysed using narrative synthesis.

**Results:**

From 59 eligible studies, 9 themes were generated: (1) affordability and accessibility; (2) autism‐related factors and cognitive or motor skill differences; (3) the dental environment; (4) managing CYP's behaviour; (5) responding and adapting to the needs of the autistic CYP and their parent/carer; (6) attitude of dental health professionals (DHPs) towards autistic CYP and their parents/carers; (7) knowledge of how to care for and support CYP's OH; (8) empowerment of parents/carers and collaboration with DHPs; and (9) communication and building rapport.

**Conclusion:**

The adoption of healthy OH behaviours and access to dental care by autistic CYP is impacted by a range of factors including those intrinsically related to a diagnosis of autism, for example, communication and those often associated with autism, for example, sensory sensitivities. Access to better OH and dental care can be facilitated by responding to the individual needs of autistic CYP through accommodation, education and adaptation. This necessitates greater awareness and knowledge of autism amongst DHPs and the provision of appropriate services. More methodologically robust intervention studies are needed to identify effective ways to support autistic CYP in achieving good OH and access to dental care.

**Patient and Public Contribution:**

The review protocol was developed with members of the project patient and public involvement group who provided the autistic voice, contributing to the interpretation of the review findings and writing of the manuscript.

## 1 BACKGROUND

Children's oral health (OH) matters. Poor OH in childhood can cause pain, discomfort and social embarrassment and impacts on quality of life and long‐term OH.^1^ Most OH problems such as dental caries and periodontal (gum) disease are largely preventable. However, poor OH is common, with vulnerable and socially disadvantaged people disproportionately affected.^2^ This reflects a range of interacting factors at the individual and societal levels.^2^ One group that experiences significant unmet needs in relation to OH is autistic children and young people (CYP).^3^

Autism is a neurodevelopmental condition that is diagnostically characterized by persistent difficulties with social interaction and communication, and restricted, repetitive patterns of behaviours.^4^ Autistic people may also experience altered sensory responsivity across all senses. Autism is a spectrum that presents in a range of different ways such that there is no typical autistic child and no one‐size‐fits‐all health care appropriate for everyone.^5^

Autistic CYP have a high prevalence of dental caries and periodontal disease and poor oral hygiene levels,^6^ with studies consistently showing high levels of unmet treatment needs.^3^ They are also more likely than neurotypical CYP to receive treatment under general anaesthesia.^7^ There are several reasons why the risk of dental disease may be higher in autistic children. They often have a restricted diet due to sensory sensitivity around taste, texture and smell. They may experience avoidant/restrictive food intake disorder, pica and the need for ‘sameness’, which can mitigate against achieving a balanced diet.^8^ Other factors include the use of tricyclic medications (a common side effect of which is dry mouth) and poor oral hygiene.^9^ The latter may arise because sensory sensitivities and issues around manual dexterity can impact the ability of autistic CYP to brush their teeth.^10^ Some CYP may not have the skills or knowledge to care for their own teeth effectively and are dependent on others to provide this care.

Autistic people encounter difficulties in accessing dental care. Attending dental visits can be a stressful experience for autistic CYP and their families.^7^ Sensory sensitivities, difficulties relating to social interaction and communication and resistance to change can make dental visits unpleasant for autistic CYP. This may be reflected in their behaviour, which can pose challenges for the dental team. A lack of awareness, education and training about autism among dental health professionals (DHPs), together with the behavioural distress that autistic patients may experience, contribute to the reluctance of some DHPs to treat autistic CYP.^11^, ^12^

The unmet OH needs of autistic CYP must be seen in the context of the wider health, social and economic inequalities that autistic people experience across the lifecourse. Autistic CYP and adults endure unmet health needs and inequality in access to health care that affects their mental and physical health, unnecessarily reducing life expectancy.^13^

The aim of our research is to collate the evidence on the factors that influence OH behaviours, access and delivery of dental care to autistic CYP. The term ‘oral health behaviours’ here refers to behaviours to maintain OH and prevent disease as identified by Public Health England.^14^ These include toothbrushing, using fluoridated toothpaste and frequency of sugary food and drinks. In consultation with the study's patient and public involvement (PPI) group, we have adopted identity‐first language (e.g., autistic children). This is widely used by the autism community and is the language preference of choice of the National Autistic Society UK. We define ‘young person’ as being a person aged 10–19 years.

## 2 METHODS/DESIGN

The protocol for the systematic review is published^15^ and registered with PROSPERO (reg. number: CRD42021248764).

### 2.1 Eligibility criteria

Eligibility criteria are shown in Table 1. Research from countries with a very high Human Development Index (HDI) only was included in the review (for a list of countries and an explanation of HDI, see Appendix 1); this facilitates comparability and supports the transferability of findings to countries with advanced health care systems. Studies published in any language were included. Studies in French and Japanese were translated by the authors. There was no restriction on the date of publication.

**Table 1:** Summary of inclusion and exclusion criteria

Study characteristic	Inclusion criteria	Exclusion criteria
Population	Autistic children and adolescents 19 years of age or younger at the time of the study. Studies addressing both adults and children were included if the data/findings relating to children were clearly defined and reported separately.	Autistic individuals aged 20 years and older at the time of the study.
	Parents/guardians/caregivers, support staff (e.g., support workers, volunteers, teachers), who must be caring for, working with or supporting at least one autistic child or adolescent.	
	Dental health professionals to include all individuals involved in the provision of dental care or promotion of oral health, for example, dentists, dental hygienists, dental nurses, oral health educators and other members of the wider dental team such as receptionists.	
Setting	Studies from countries with a Human Development Index value of 0.8 or above.	Studies from countries with a Human Development Index value below 0.8.
Outcomes	Studies that include outcomes relating to:	Studies that do not include outcomes relating to the factors influencing oral health behaviours in autistic children and adolescents, their access to dental care or the provision of dental care to them.
	Factors influencing oral health behaviours in autistic children and adolescents.	
	Factors influencing access to dental care services by autistic children and adolescents.	
	Factors influencing the provision of dental care to autistic children and adolescents by dental health professionals.	

### 2.2 Information sources and search strategy

Literature search strategies were developed by an information specialist (L. B.) using subject heading and synonyms for autism and dental care or OH. The databases Embase, Web of Science, Dentistry & Oral Sciences Source, MEDLINE, PsycINFO, Scopus, CINAHL and SocINDEX were included. Grey literature^16^ was identified by an internet search using the search engine Google, established sources of grey literature and websites of relevant organizations including EThOS, Social Care On‐line, Public Health England, Health Foundation, British Society for Disability & Oral Health, British Society for Paediatric Dentistry, National Autistic Society, British Dental Association, Autistica, National Institute for Health and Care Excellence and Mencap. The focus on UK‐based grey literature reflects the fact that this review is part of a wider study that aims to inform UK policies and practices in relation to the OH care of autistic CYP. Database and grey literature searches were conducted in February 2021 and repeated in November 2021 (see Appendix 2 for search strategies).

### 2.3 Selection process

Search results were uploaded to Rayyan,^17^ a systematic review web application, for screening. Two reviewers (J. E. and M. P.) independently screened all titles and abstracts against the inclusion and exclusion criteria. Full texts of potentially relevant articles were retrieved and independently screened by J. E. and M. P. Where the two reviewers disagreed on article inclusion, consensus was reached by discussion with the third reviewer (R. W.).

#### 2.3.1 Data extraction

Table 2 shows the data items extracted for quantitative, qualitative and mixed‐methods studies. For qualitative studies and the qualitative components of mixed‐methods studies, extracted data also included themes related to the outcomes of interest.

**Table 2:** Data items

	Data Items
Authors	Children's oral health behaviour and dental visits
Year of publication	Barriers to oral health
City/country	Access to dental care
Aim of study	Provision of dental care
Type of study	Facilitators for oral health
Study design	Access to dental care
Data collection method	Provision of dental care
Recruitment	Description of intervention
Outcome measure	Study details
Sampling	Country
Analysis method	Study methodology
Dental care setting	Data collection methods
Oral health setting	Participant characteristics
Oral health behaviour	Dental care setting
Participant population	Oral health setting
Participant characteristics	Outcome of interest
Children's diagnosis	

### 2.4 Assessment of methodological quality

The studies were critically appraised using the Mixed‐Methods Appraisal Tool (MMAT).^18^ This is a critical appraisal tool designed for systematic mixed study reviews that permits appraisal of the methodological quality of five study categories: qualitative research, randomized‐controlled trials (RCTs), nonrandomized studies, quantitative descriptive studies and mixed‐methods studies. Specific appraisal questions relate to each study category. The appraisal was carried out by J. E. and M. P.; any disagreements were resolved through consensus. Given the lack of consensus on the use of quality appraisal results in qualitative research synthesis,^19^ all 59 studies that fulfilled the inclusion criteria were included in the analysis regardless of their quality assessment score. We use percentages descriptors to indicate the score for each domain and the quality criteria fulfilled (Table 3).

**Table 3:** Measures of data quality and vote counting

Data quality score criteria
100%—All 5 quality criteria fulfilled for domain
80%—4 out of 5 quality criteria fulfilled for domain
60%—3 out of 5 quality criteria fulfilled for domain
40%—2 out of 5 quality criteria fulfilled for domain
20%—1 out of 5 quality criteria fulfilled for domain
0%—0 out of 5 quality criteria fulfilled for domain

### 2.5 Data synthesis

Quantitative and qualitative data were synthesized together through data transformation using a convergent integrated approach, where all the included studies were analysed using the same methods and with the results presented together.^20^ To enable this, data from quantitative studies and the quantitative aspects of mixed‐methods studies were extracted and converted into textual descriptions (qualitized). The extraction was led by the research questions and was carried out in a systematic way, extracting the same information for all studies. These textual descriptions were then integrated with the data from qualitative studies for analysis. The narrative synthesis drew on the framework and techniques described in ‘ERSC Guidance on Conducting Narrative Synthesis’.^20^

Thematic synthesis,^21^ conducted by J. E. and M. P., was used to carry out an inductive analysis of the qualitative data and the ‘qualified’ quantitative data from the studies. The data were coded, and codes sharing common meaning or experience were grouped. The main, recurrent or most important descriptive themes in the literature were identified. The constant comparative method was used to ensure translation of concepts from one study to another, looking for the similarities and differences between the findings reported in the papers/reports. Analytic themes were created by exploring whether the various descriptive themes led to a new interpretation of the findings not explicitly stated in the primary studies. The ENTREQ^22^ and PRISMA guidelines^23^ were followed for the reporting of the review.

## 3 RESULTS

**Figure 1:**
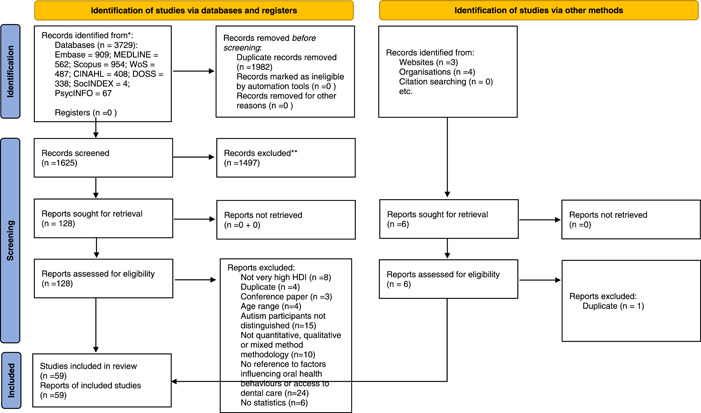
PRISMA diagram.

The flow of information is shown in the PRISMA diagram (Figure 1). Fifty‐nine studies were identified for inclusion, 56 from peer‐reviewed journals and 3 postgraduate theses.^24^, ^25^, ^26^ There were 42 quantitative, 11 qualitative and 6 mixed‐methods studies, of which 18 were intervention and 41 were descriptive studies. The study characteristics are presented in Appendix 3. The studies came from 13 HDL countries, the majority from the United States of America (44%). Twenty‐three were nonrandomized studies and three were pilot RCTs. Sixty‐three percent of studies used surveys or questionnaires; other data collection methods included interviews, focus groups, record reviews, oral examinations and physiological tests. Participants were recruited from schools, autism support groups and services, hospitals, private dental services and others. In 65% of studies, participants were parents/caregivers, in 23%, CYP were the sole participants and in 15%, DHPs were included as participants.

### 3.1 Factors influencing OH behaviours, access to and provision of dental care to autistic CYP

Themes identified from the analysis of the descriptive data reporting the perspective and experiences of the CYP, parent/carer or dental care professional are presented in Table 4. Table 5 presents representative quotes that illustrate these themes.

**Table 4:** Themes from studies

Themes	Factors relating to the autistic children's/adolescents' willingness and ability to adopt good oral behaviours and access dental care	Factors relating to parent's/carer's willingness and ability to facilitate their child's adoption of good oral health behaviours and access to dental care	Factors relating to dental care teams willingness and ability to support adoption of good oral health and accommodate the needs of autistic children/adolescents
Affordability and accessibility		Cost (insurance); time; travel;	Funding and financial reimbursement (dental contract/specialist services); time/costs
Autism‐related factors and cognitive or motor skill difficulties	Sensory sensitivities;	Caring for the child's non‐oral health needs;	
	Difficulty in changing routine; other nonautism‐related motor skill/cognitive differences;		
		Competing priorities;	
		Ability to interpret and communicate the child's needs and feelings.	
	Ability to communicate feelings and needs.		
The dental environment	Challenging environment for the child—waiting room, waiting times, dental office and procedures	Support of the child in challenging dental environment	Providing an inclusive dental environment
Managing children's behaviour	Stress, anxiety and distress in the dental environment.	Lack of social support at home;	Managing the child's distressed behaviour
		Managing the child's distressed behaviour in the dental environment; managing behaviour/distress after the dental visit for the self, child and family.	
Responding and adapting to the needs of the autistic child and parent/carer		Preparation and practice.	Providing the parent and the child with strategies to help support good oral health;
			Flexibility and adaptation of processes in dental practice to the needs of each individual child;
			Preparation and practice.
The attitude of DHPs towards autistic CYP and their parents/carers		Finding a ‘suitable’ dentist	Dental team's understanding of the challenges faced by parents of autistic children and adolescents and their parents;
		Felt criticism—being judged by dental professionals.	
			Acceptance of difference
Knowledge of how to care for and support the child's oral health	How to use a toothbrush;	Knowledge of and attitude towards oral health;	Dental team's knowledge and experience of treating/managing autistic children;
	Previous experience of dental care;	Implementing strategies to support the child's oral health;	Knowledge of ways to make the dental experience easier for the child, parent and dentist.
	Knowledge of ways to make the dental experience easier.		
		Previous experience of own and child's dental care;	
		Knowledge of ways to make the dental experience easier for the child and parent.	
The empowerment of parents/carers and collaboration with DHPs	Voice of the child.	Confidence to advocate for the child.	Parent engagement;
			Recognition and use of parental knowledge of the child and strategies.
Communication and building rapport		Communication and rapport with dental team.	Communication between the dental team and the child; Establishing rapport between the child, parent and dental team.

Abbreviations: CYP, children and young people; DHP, dental health professional.

**Table 5:** Quotes illustrating themes relating to factors influencing oral health behaviours, access to and provision of dental care

Themes	Relevant quotes from qualitative descriptive studies
Affordability and accessibility	Parent—‘I was finally able to find a paediatric dental provider who was wonderful and [had] patience. However, our dental insurance would not cover the cost of that provider, as being a specialist; the insurance found it an unnecessary expense’. (Hauschild et al., 2019)
	Parent—Parent in a US study describing how, as soon as she thought her son had ASD, she ‘…changed my insurance from a HMO to a PPO because I knew if I stayed in the HMO it was going to be really hard to get a child‐centred dentist, much less one with experience with special needs kids’. (Stein Duker et al., 2017)
Autism‐related factors and cognitive or motor skill difficulties	‘I was totally unable to get into her mouth for the first several years of her life because she was so sensitive. She is also not communicative so it doesn't help to explain’. (Lewis et al., 2015)
	‘This was difficult. This was very difficult. We didn't use toothpaste for a long time. He had a very hard time with the taste. The taste was not appealing to him’. (Abomriga, 2017)
	‘Brushing teeth falls at the bottom of my priority list. There are so many stressors. We're all exhausted by the end of the day’. (Lewis et al., 2015)
	‘Due to his condition, we have to do a lot of things for him. So, the checklist is long and brushing teeth comes at the very end. Since both of us are working, by the time we completed the entire checklist, we wear ourselves out’. (Rohani et al., 2018)
Managing children's behaviour	Parent—‘My son is very sensory oriented… once he steps in that environment he feels uncomfortable… all the sensory devices will just make him so uncomfortable’. (Stein Duker et al., 2017)
	Parent—‘My son couldn't stand being touched. He was unable to follow multiple steps’.
	‘It was difficult because my child doesn't like to sit in the chair and doesn't like when the light is on’.
	‘She has so many sensory issues the light, the noise, and even the little things bug her…’. (Hauschild et al., 2019)
	Parent—‘…she'll usually be hyper on the way there, very hyper…and then as soon as we get there…she will just be back and forth to the toilet…often, her name will be called and she's in the toilet…she's just very stressed. Very, very anxious, very worried about what they're going to do’. (Thomas et al., 2018)
	Dentist: ‘Someone with ASD doesn't understand that you want to take care of his mouth and that it's a good thing that you scratch with a scaler, with a sound‐producing, rotating machinery that develops vibrations and sounds, which perhaps by someone with ASD is experienced much stronger and more exciting than average people. This person sees it as a burden not as a possible benefit. So it scares them and they find it unpleasant, therefore they will repel’. (Koojiman, 2016)
The attitude of DHPs towards autistic CYP and their parents/carers	Parent—‘There was a lot of eye rolling. People look at you like why can't you just discipline your kid out of it. They never explained anything to him…; the staff wasn't really trying, they weren't very warm or caring and they just gave up’. (Hauschild et al., 2019)
	Parent—‘There have also been times when I felt like the hygienist would talk down to me because my son had a cavity, and really questioned how I enforce his brushing habits at home’. (Hauschild et al., 2019)
	Parent—‘There's quite a snotty receptionist there’. I don't think she's at all child‐friendly to be fair…. the slightest squeak that [my child] makes and she's on the phone and she's like, ‘oh. Um. I'm sorry. I can hardly hear you, we've got some children in here, and they're being a bit naughty’. (Thomas et al., 2018)
	Parent—‘…A lot of positive reinforcement is helpful to my son, but I'm also going to throw in that it is helpful to me. It's important for me to see that the dentist is sensitive to my son and the way that it is and it just it doesn't faze her [the dentist] the same way it would someone else. Like he [my son] could be like completely melting [down] right there having like wanting to get out and you know it's embarrassing or like it gets very tense and to see her [the dentist] just be understanding and not change and just keep at it and keep her positive reinforcement’. (Stein Duker et al., 2019)
	Parent—‘I don't think she had any dealings with autism before that. I'm not sure she has now, you know, since. But I just think it was her open attitude and the fact that we said, ‘look, is this… you know, we want to do this, is this ok?’ and she was like, ‘yeah, absolutely’. So, it was her, completely her attitude, you know’. (Thomas et al., 2018)
Knowledge of how to care for and support the child's oral health	Parent—‘It was a struggle to brush his teeth before. But after I met a dentist at the hospital, he taught me how to brush the back teeth. My child is now more cooperative during tooth brushing’. (Rohani et al., 2018)
	Parent—‘We made an appointment for him to see that dentist. But she did not have any experience with special needs at all. So, she wasn't very good at handling him. He couldn't sit on the chair. He would run around the office. Just waiting at the waiting room…that is just not a very good experience for anyone in that way’. (Abomriga, 2017)
The dental environment	Parent—“Because the waiting room is a whole separate thing – it's almost like having an appointment in its own right, going and sitting in the waiting room – that's a thing, and then you go and do the dentist which is another big thing.” (Stein Duker et al., 2019)
Responding and adapting to the needs of the autistic child and their parent/carer	Parent—‘I presented a whole thing at the school on toothbrushing at school so they would start to do that at school. I did because it was such a struggle at home, and I wanted someone else to be working on it, too, and for him to see his peers doing it’. (Lewis et al., 2015)
	Parent—‘I am really pleased with the way our dentist works. The dentist started with a really, really slow routine to make him comfortable. The chair goes up and down. My son loves water and he loves to suck it up in the silly straw, and then they said, “let's look in your mouth and count your teeth”. The first time they didn't get any cleaning done…it is still really hard to get his teeth cleaned’. (Lewis et al., 2015)
	Parent—‘What helps my son is social stories, preparing him using a book I created called Going to the Dentist, with actual pictures of the dentist's office and the people he is going to see’. (Lewis et al., 2015)
	Parent—‘with routine suddenly things will click…it is a big problem if she feels out of control and doesn't know what's coming next and negative experience is such a setback’. (Parry and Shepherd, 2018)
The empowerment of parents/carers and collaboration with DHPs	Parent—‘I want them to ask: “what is the best way to proceed with your son?”’.
	Parent—‘No one has really ever asked me, but I would be thrilled if someone wanted to know if there are special things we need to do differently because of his autism’. (Lewis et al., 2015)
	Parent—‘Well, I suppose it's more of “we”, as parents need to actually give them what works for our child or what our child – because with the autism and Asperger's, they're all so different – to have a blanket, “well this is what you need to do” I suppose is quite hard to do, but I mean, maybe it's more, the practice is saying “well let us know what we can do for you – what do you think is going to work for you?”’. (Thomas et al., 2018)
Communication and building rapport	Parent—‘My son was unable to tell me if and when he had a toothache, or a blister, or anything else in his mouth’. (Hauschild et al., 2019)
	Parent—‘They spoke to him in a very normal way. They didn't appear to be worried that he may not understand or not respond or do things as quickly as they wanted to. I actually found the experience was fantastic and the staff were very good in the way they managed someone like L. who has special needs’. (Taghizadeh et al., 2019)
	Dentist—‘The bottleneck for me is that you don't get the usual feedback on direct contact. So you try to make contact and you try to get response so that you can continue to the next initiative, but in people with ASD it's often the case that you will not get the expected response and therefore you get lost in the moment. And not only you are lost as practitioner, but also the patient doesn't understand you and that's a big challenge’. (Koojiman, 2016)
	Dentist—‘You often see when it's not clear what will happen, that someone who doesn't get all the pieces together will express himself with repetitive behaviour or defensive behaviour, because he is anxious since he doesn't understand what will come’. (Koojiman, 2016)
	Dentist on establishing rapport—, … ‘it's been very difficult when they've been four, five, six, or seven, and trying to manage them, but you know, they've kept coming back, they've been OK to come back and then they suddenly change and they become a bit more accepting of the treatment and they are still coming in and by the time they're ten, eleven, twelve, they love it!’. (Parry and Shepherd, 2018)

#### 3.1.1 Affordability and accessibility

Ten studies referred to the financial cost of dental treatment and ineligibility for dental health insurance as barriers to dental care access.^24^, ^27^, ^28^, ^29^, ^30^, ^31^, ^32^, ^33^, ^34^, ^35^ Of these ten studies, six were from the United States of America, two were from Saudi Arabia, one was from Canada and one was from Hong Kong. All these countries fund paediatric dental care through a mix of publically funded programmes and private insurance. Distance and travel time^32^, ^36^, ^37^ result in direct and indirect costs. There are also financial issues for providers. In a survey of US dentists,^38^ 72.3% agreed that financial compensation for treating autistic patients was inadequate and not reflective of the increased costs arising from the need for longer appointments for familiarizing/desensitizing. Dentists must reconcile the need to offer time to families and financial restraints.^39^ In the United Kingdom, DHPs felt the complexity of managing the dental care of autistic children and the time required is not recognized by the United Kingdom state‐funded dental system^12^, ^40^ and expressed concerns regarding the closure of specialist services due to a lack of funding.^12^

#### 3.1.2 Autism‐related factors and cognitive or motor skill difficulties

Autistic people may experience under‐ or oversensitivity to light, sound, taste or touch. Sensory sensitivities were cited by nine studies as a barrier to beneficial OH behaviours.^24^, ^25^, ^34^, ^37^, ^41^, ^42^, ^43^, ^44^, ^45^ Changing routines to include OH care can also be challenging.^25^ Some autistic CYP also have cognitive and motor skills difficulties that can affect their ability to carry out OH behaviours and depend on parents/carers to help them maintain oral hygiene.^46^ Parents/carers may have to help their autistic children with a range of daily living tasks and can be overwhelmed when meeting their children's needs, such that OH care falls low on the priority list.^41^, ^46^ Sensory sensitivities,^11^, ^25^, ^26^, ^28^, ^31^, ^34^, ^36^, ^37^, ^40^, ^41^, ^42^, ^43^, ^44^, ^45^, ^46^, ^47^, ^48^ cognitive and physical challenges^33^, ^49^, ^50^ and the degree/level of required support and adjustment^49^ were all identified as barriers to dental care access. Children may also experience fear and/or dental anxiety.^11^, ^26^, ^42^, ^43^, ^48^, ^51^, ^52^ In a US study, 52% of parents of autistic children reported difficulty with three or more sensory variables compared to 6% of parents of typical children (*p *< .001).^48^ Sensory sensitivities also impact on the provision of dental care.^12^, ^25^, ^26^

#### 3.1.3 The dental environment

The environment of the dental clinic, the lights, sounds, tastes, smells, the movement of the dental chair and the proximity of the dental professional when carrying out an examination can all have an impact on the autistic child. This can make a visit to the dentist an unpleasant and challenging experience.^31^, ^42^, ^48^, ^39^ Ten studies cited the waiting room and dental office environment^11^, ^28^, ^31^, ^36^, ^37^, ^41^, ^42^, ^44^, ^46^, ^51^ as barriers to access. In a UK study,^51^ 78.5% of parents reported that waiting too long in the waiting room impacted their child's behaviour when they see the dentist. Parents suggested that the use of child‐friendly environments in the waiting room and the dentist's office, such as video games, toys and creating small spaces for children within the waiting room, can make the dental visit more accessible.^34^

#### 3.1.4 Managing children's behaviours

Difficulty in managing children's behaviours, including non‐co‐operation and compliance with dental procedures, was one of the most frequently reported barriers to dental care access cited by parents and dentists.^25^, ^27^, ^28^, ^30^, ^32^, ^33^, ^35^, ^36^, ^37^, ^42^, ^43^, ^46^, ^48^, ^53^, ^54^, ^55^, ^56^ A US study^33^ found that autistic children with more behavioural difficulties had an increased odds of unmet dental needs compared to those with autism who showed behaviours similar to same‐aged peers (odds ratio: 3.35, 95% confidence interval: 1.69, 6.67). The uncooperative behaviours of some autistic children affect the ability of practitioners to carry out the dental procedures needed to care for their teeth,^25^ with obvious implications for OH.^30^

#### 3.1.5 Responding and adapting to the needs of the autistic child and their parent/carer

Factors influencing provision include responding to individual needs.^18^, ^38^, ^50^, ^67^ Parents/carers perceived that the lack of flexibility and adaptation to individual needs^12^, ^26^, ^31^, ^39^, ^40^, ^57^, ^58^ including long waiting times^11^, ^32^, ^36^, ^37^, ^58^ and lack of continuity^11^, ^24^ impeded their ability to access dental care for their child.

Parents suggested procedures such as phoning the dental clinic before their arrival and permitting immediate access to the dental surgery.^36^ Other flexible ways of working and adaptations supported by both parents and dentists include the scheduling of appointments to shorten waiting times,^38^, ^39^, ^40^ sensory strategies,^39^ special visual aids for communication,^38^ rewards^26^, ^38^, ^39^ and allowing parents to accompany their child.^38^ Staff continuity is important, particularly for children who struggle with changes in routine.^11^, ^24^

A UK study^51^ evaluated a real‐time text messaging service to improve dental attendance experience. The majority of parents/carers believed that the intervention reduced anxiety and enabled their CYP to be more accepting of dental treatment, but the sample size was small (*n* = 17).

Adjustments reported by parents and dental staff include being cautious about the use of loud tools, avoiding products with strong tastes, using support to help with bright lights and loud noises, computers to distract the children and inviting patients to bring special objects from home to help with anxiety.^58^ A nonrandomized study^59^ (*n* = 28) evaluated the effectiveness of audio‐visual distraction in modifying children's behaviour during dental procedures and observed a significant reduction in the mean heart rate (*p *< .02) during dental screening. A pilot RCT^60^ examined the feasibility and benefit of using two types of electronic screen media to reduce fear and uncooperative behaviours in autistic children undergoing dental visits. There were significant decreases in the mean anxiety and behaviour scores (*p* = .03), but the study was not powered to detect significant differences between the intervention and control groups.

A small (*n* = 44), nonrandomized case‐control study piloted a sensory adapted dental environment to reduce distress, sensory discomfort and perception of pain in autistic children undergoing oral prophylaxis.^61^ There was a significant increase in the proportion of autistic children rated as relaxed and co‐operative (*p *= .08).

Seven descriptive studies highlighted the benefits of preparation and practice for facilitating access and the successful delivery of dental care.^12^, ^38^, ^39^, ^40^ Providing information to the patient and their family on what to expect at the dental visit can help to alleviate stress.^11^ Social stories, books, take‐home practice packs, visual training and tell‐show‐do strategies^36^, ^39^, ^58^, ^62^, ^63^ can help prepare the child and their family for the visit, reduce anxiety and increase acceptance of oral examinations. A pilot RCT tested whether a pictorial cue board, designed to show the steps involved in a dentist visit, could help autistic children have successful routine dental cleaning visits.^64^ The intervention indicated potential benefits, but was too small (*n* = 14) to derive any conclusions.

A feasibility study (*n* = 59)^65^ used a multidisciplinary approach using IT, behavioural techniques and a clinical protocol to familiarize autistic children to the dental setting and procedures. It showed significant positive changes (*p *< .001) in parents' responses to questions about their child's ability to brush their teeth and carry out other oral hygiene activities. Another small study (*n* = 44)^66^ evaluated a programme of familiarization and repetitive tasking. Patients showed a significantly greater improvement in behavioural scores compared to the control group (*p *= .03) and had significantly lower referrals for dental treatment under general anaesthesia (*p *= .04). Nelson et al.^67^ evaluated the effectiveness of a dental desensitization programme for autistic children (*n* = 168) and examined what characteristics were associated with a successful dental examination. A total of 77.4% of children achieved a minimum threshold exam (MTE) within 1–2 visits. A follow‐up study^68^ indicated that 92% of children who had achieved an MTE at baseline maintained this ability at Year 2.

Modelling expected behaviour at a visit to the dentist by the parent and other members of the family is useful.^26^ The modelling of toothbrushing at the dentist and at school,^25^, ^41^ behavioural techniques including positive re‐enforcement^24^, ^46^ and incorporating it into the daily home and school routine^41^, ^69^ can help children adopt beneficial OH behaviours.

#### 3.1.6 The attitude of DHPs towards autistic CYP and their parents/carers

The attitude of DHPs and the wider dental office team^11^, ^28^, ^31^, ^32^, ^39^ towards autistic children influences access to dental care. Parents' perception of being negatively judged is a barrier to access.^11^, ^24^, ^40^, ^41^, ^70^ Parents described how they felt criticized for not sufficiently controlling or disciplining their children^11^, ^31^ and judged for the state of their children's teeth. This made the dental visit more stressful.^11^ Difficulty in finding a ‘suitable’ dentist was reported in 14 studies.^11^, ^27^, ^28^, ^29^, ^31^, ^32^, ^33^, ^34^, ^35^, ^37^, ^39^, ^48^, ^55^, ^70^ By a ‘suitable’ dentist, parents mean dentists who have a positive attitude towards treating autistic children and who accept autistic children for treatment.^39^, ^58^ A positive attitude facilitates positive reinforcement and deescalates what can be a tense and difficult time for the child and the parent.^39^, ^58^

Identifying a dentist with adequate knowledge of autism and experience with children can be challenging. In a study from Saudi Arabia,^27^ 54% of the participants reported difficulty in locating an appropriate dental clinic to treat their autistic child and 32.7% reported that dentists refused to care for their children. This is echoed in US studies^39^, ^48^

#### 3.1.7 Knowledge of how to care for and support the child's OH

Parental OH education^46^, ^71^ and education of the child in toothbrushing techniques^25^ act as facilitators for the adoption of beneficial OH behaviours and help the child to better tolerate oral care. Two studies^72^, ^73^ assessed the effectiveness and feasibility of a digital dental education programme for autistic children and their caregivers. A pilot feasibility study^72^ found improved oral hygiene in both cases and controls, but showed high attrition rates. Another study (*n* = 15)^73^ found a significant increase in the mean oral hygiene knowledge scores in children and parents at 4 weeks postintervention. A feasibility study (*n* = 12)^74^ evaluated the acceptability and effectiveness of a mechatronic brush to help children (autistic and nonautistic) brush their teeth and showed improvement in toothbrushing motions and reduction in stress. Two studies^75^, ^76^ used social stories to teach toothbrushing skills to preschool autistic children. One (*n* = 181) showed a significant improvement (*p *< .001) in toothbrushing performance, oral hygiene status and gingival health status.^75^ The other study^76^ showed an increase in the percentage of children brushing once or twice a day with fluoride toothpaste, but was too small (*n* = 32) to show statistical significance.

A study^77^ assessed a training programme using digital iPad applications for teaching autistic CYP (*n* = 52) to be compliant with a dental examination. It showed positive statistically significant improvements (*p *< .01). Another study^78^ assessed the effectiveness of a psychoeducational intervention programme to facilitate the performance of a series of steps of oral examination in autistic CYP (*n* = 74). It showed a significant increase in the number of oral assessment steps (*p *< .0001) and behaviour scores (*p *< .0001).

Fourteen studies cited the dental team's lack of knowledge and experience of the dental treatment needs and care of autistic CYP as a factor influencing access.^12^, ^24^, ^25^, ^28^, ^31^, ^32^, ^37^, ^38^, ^39^, ^40^, ^41^, ^46^, ^57^, ^58^ DHPs reported a lack of knowledge regarding the care of autistic children^25^, ^38^, ^47^, ^79^ and emphasized the need for dental education and training.^12^, ^39^, ^40^, ^58^, ^79^ In a UK study of DHPs,^12^ less than half (44.7%) had received training in autism. Respondents were only ‘somewhat confident’ in their ability to treat autistic patients and were least confident in knowledge of relevant local care pathways/services for autistic people.

#### 3.1.8 The empowerment of parents/carers and collaboration with DHPs

Parents want the dentist or staff to ask in advance how, specifically for their autistic child, the visit can be made to go as smoothly as possible. Some parents may not feel empowered to speak up about their children's needs and preferences or feel that the dental team do not want or value their views.^11^, ^52^ When DHPs adopt a collaborative approach, they can draw on the parents' knowledge of the child, their individual needs, potential sensorial triggers and reactions^11^ and can use some of the strategies that the family uses at home to support successful visits and to identify types of positive reinforcement and external motivators for individual children.^11^, ^39^ Such engagement with parents can have a positive impact on dental care.^11^, ^12^, ^39^, ^41^, ^58^, ^80^

#### 3.1.9 Communication and building rapport

Communication between dentists and autistic children and their parents is a key factor in access to successful dental care.^12^, ^25^, ^26^, ^33^, ^50^, ^52^, ^57^, ^58^ Autistic children commonly experience difficulties in social interaction and communication. Some children have difficulty communicating their pain and how they are feeling during treatment sessions to their parents or the dental team. Some dentists and other members of the dental team find it difficult to communicate with autistic children and adolescents and to make, maintain or restore contact.^12^, ^25^, ^26^ In cases where the child's verbal communication is limited or absent and/or their level of understanding is lower, this can present particular challenges. In one study,^33^ autistic children with additional communication (or physical function) difficulties were more likely to have unmet dental needs compared to other autistic children who did not have these difficulties (*p *= .05). Studies indicate that communication during the examination needs to be age appropriate and unambiguous, giving children time to process and understand information and providing opportunities for questions from the child and their parent/carer.^11^, ^12^, ^25^, ^26^ Good communication was characterized by parents and DHPs as being open and clear, with good listening skills contributing to the development of a rapport between all parties and facilitating trust between the child, their parents and the dental professional.^11^, ^12^, ^24^, ^26^, ^27^, ^39^, ^40^, ^58^ This building of rapport can be viewed as an investment for all concerned.^40^

## 4 DISCUSSION

This mixed‐methods narrative review systematically explores the available literature on the factors influencing the OH behaviours of autistic CYP, their access to dental care and how dental care is provided to them. It highlights the challenges and enablers to the adoption of good OH behaviours and the use of dental care services from the perspective of autistic CYP, their parents/caregivers and DHPs.

The factors that affect the ability of autistic CYP to adopt beneficial OH behaviours that were most frequently cited in this review were sensory sensitivities, factors relating to cognitive and motor functions and competing parental priorities. Other barriers that a recent review report are also experienced by nonautistic CYP, and include lack of OH knowledge and child behaviour management.^81^ Facilitators relevant to both autistic and nonautistic CYP include increased parental OH knowledge, the adaption of the social environment to facilitate parental supervised toothbrushing and positive attitudes towards OH.^81^ In addition, our review identified the modelling of good OH behaviours by the family and peers, incorporation of toothbrushing into the daily school routine and the use of behavioural techniques such as positive re‐enforcement as ways in which the adoption of beneficial OH behaviours can be enabled.

A range of different factors were found to impede the ability of autistic CYP to access dental care. In keeping with the findings from other reviews looking at vulnerable children's access to dental care,^81^, ^82^ finding a suitable provider, inadequate insurance cover, parents'/carers' lack of access to OH information, lack of professional knowledge and training and failure to fully accommodate the needs of the child were the most frequently cited barriers to care. Sensory sensitivities, the child's reaction to the dental environment and difficulties in managing the child's subsequent behaviour were also frequently cited as barriers. Communication and the attitude of the dental team can act as barriers or enablers to access. Dental professionals' and parents' support of preparation and practice through social stories, desensitization/familiarization and family modelling can facilitate access. The studies in this review most frequently cited dental education and training in the care of autistic children and the use of parent's knowledge of their child and their autism as enablers to the delivery of dental care to autistic CYP.

The intervention studies all showed a positive trend towards the intervention, but were generally of poor quality (e.g., failure to report methodological detail, lack of control groups, a lack of control for potential confounders) and/or had very small sample sizes. With only a few exceptions,^75^ this precluded any robust conclusions being made about the impact of these interventions on the OH behaviours of autistic CYP, their access to dental care or on the delivery of care. A Cochrane review of oral hygiene interventions for people with intellectual disabilities^82^ that included autistic children concluded that the clinical importance of interventions was unclear, with the evidence of mainly low or very low certainty. Given the importance of establishing and maintaining good oral hygiene and access to dental care, and the degree to which this is a matter of concern to parents/carers of autistic children, it is surprising that the number of intervention studies looking at these issues is relatively low and the quality is poor. There is a clear need for more robust studies to establish how best to support autistic CYP and their families/carers to establish and maintain good OH.

The waiting room emerged from the review as a challenging, anxiety‐inducing space for autistic CYP. Saqr et al.^83^ identified the waiting room and waiting time as significant barriers to primary care access for autistic adolescents that detrimentally impact the patient's ability to access care. Implementing changes in processes to allow for a reduction in waiting time, for autistic CYP to be seen at less busy times and for the provision of items for soothing or distraction could help to make the experience less anxiety‐inducing. Despite the importance of these issues, only one study in this review trialled an intervention targeted at process (to reduce waiting times).^51^

Difficulty in coping with unfamiliar sensory experiences, procedures and social interactions can be expressed by the child as behavioural distress that may be difficult for both the parent/carer and members of the dental team to manage. The behaviour of the autistic CYP at home or in the dental environment was identified as a theme, but it can also be seen as an important thread running through the systematic review analysis. All behaviour is a form of communication and a child's distress behaviour is an indication that they are upset, that something is not right or their needs are not being met. Many children, including autistic children, may have difficulty communicating, because they are not able to verbally describe the problem or know what to do in a situation. At these times, children communicate their feelings or needs through externalizing behaviour. A child whose needs remain unmet may use inappropriate behaviour as a way of sending a very loud message.^84^ Thus, ‘challenging behaviours’ may be a symptom of the child's oral and dental care experience, for example, long wait times and sensory reactions. DHPs, parents/carers can help the child by trying to understand the meaning behind the child's behaviour and what they are trying to communicate through it. This provides the potential to respond better and so reduce the child's need to use behaviours that challenge so as to communicate.

A lack of understanding of autism by the dental team can leave the child and parent/carer feeling judged for what is seen as inappropriate behaviour and/or poor parenting, which contributes to parental stress.^11^ Child behaviour and parenting stress can exacerbate each other.^85^ Studies in this review show that parents/carers and DHPs acknowledge the need for better training and education of the whole dental team to increase their knowledge and understanding of autism and how to appropriately respond to and meet the needs of the autistic CYP.^12^, ^39^, ^40^, ^52^, ^58^ Dental education, postgraduate and CPD training should include greater emphasis on this area.^79^ This could potentially reduce the difficulties that autistic children and their parent/carers currently experience in finding a ‘suitable’ dentist and facilitate their receiving the care they need in primary dental care settings.

Autistic CYP vary widely in how they perceive and react to the world around them. This review shows how important it is for access to dental care that OH providers identify what works for each individual child^41^ and that they offer accommodation, flexibility and adaptation of dental care procedures to meet individual needs. There are practical barriers to achieving this, not least the financial implications of the longer and more frequent appointments that may be needed to familiarize the child to the environment and the procedures. Autistic CYP have the same rights to access good OH care as nonautistic CYP. Health care systems should be responsive to their needs.

A central message from this review is that a key enabler to successful access to effective dental care is good communication and the building of rapport between the CYP, their parent/carer and the dental professional. An open, collaborative, informed relationship between the parties can help achieve the shared goal of better OH for the child. Pivotal to this is the empowerment of the CYP and the parent/carer to express their views and the DHPs' willingness to acknowledge and listen to both the voice of the CYP and the expertize of the parent.

Autistic children and their families often experience stigma.^86^ There appears to be very little research looking at this in the context of dental care, and stigma did not emerge as a strong explicit theme in this review. It is important to note that the language used in some studies displayed a lack of awareness that could be seen as stigmatizing. A striking example was an older study^76^ that referred to the inclusion of ‘mentally retarded’ autistic children, a term that is now startling and offensive. There is current research to evidence more subtle unconscious bias in the language used by dentists when talking about the treatment of autistic children.^87^

This review highlights the challenges that autistic CYP face in caring for their OH and accessing dental care. This has the potential to negatively impact their long‐term OH and increase the burden on the dental care system. Poor OH can impact on an individual's self‐confidence and be negatively judged by society. Poor OH and the failure to establish good oral hygiene routines as a child can also lead to increased burden and cost on the dental care system. If we wish to tackle OH and the wider health inequalities experienced by autistic CYP and adults, there is a pressing need to make health care professionals and the wider public more aware and accepting of how autistic people experience the world and to remove the entrenched stigma and discrimination that impacts on the health and well‐being of autistic people across the world.

The number of people diagnosed with autism has increased considerably.^88^ As the autistic population grows, we must explore approaches to better understand and fulfil the OH needs of autistic CYP. In the 59 studies included in this review, the voice of the child is almost completely absent. Only one study^40^ interviewed autistic children about their OH challenges. Future research into the OH of autistic CYP should seek to include them in a meaningful way so that it can help shape the research and lets their voices be heard. This includes gaining the views of those autistic CYP who are nonverbal and rarely included in research. Commitment is needed from policy makers, commissioners and dental care professionals at all levels to address and overcome barriers to access to good preventive and treatment‐based dental care health care for autistic CYP.

This is the first systematic review examining factors influencing the adoption of beneficial OH behaviours, access to and delivery of dental care for autistic CYP. The contribution of the PPI group to this review is one of its strengths. For example, discussions about the appropriate use of language raised awareness across the whole study team of important issues such as identity and inclusion.

A limitation of the review is the restriction of studies included to those from countries with a very high HDI. The rationale was to facilitate comparability of results; however, it is possible that this excluded factors identified in lower index HDI countries that may be relevant to countries with higher income and more advanced health care systems. The intervention studies included in the review were overall of poor quality, with the non‐randomized intervention studies scoring particularly low in the MMAT assessment. The quality of the systematic review results may have been affected by the poor quality of the primary studies.

## 5 CONCLUSION

The adoption of healthy OH behaviours and access to dental care by autistic CYP is impacted by a range of factors. This review highlights the importance of factors intrinsically related to a diagnosis of autism, for example, communication and social interaction differences and those often associated with autism, such as sensory sensitivities. Access to better OH and dental care can be facilitated by responding to the individual needs of the autistic child through accommodation, education and adaptation. This necessitates greater awareness and knowledge of autism amongst DHPs and the provision of appropriate services. This review highlights the failure of dental care systems in countries with a very high HDI to accommodate the needs of autistic CYP. It emphasizes the need for more methodologically robust intervention studies to identify effective ways to support autistic CYP in achieving good OH and access to dental care and to improve the OH of this vulnerable population.

## AUTHOR CONTRIBUTIONS

Jo Erwin, Martha Paisi, Robert Witton and Sarah Neill were involved in the conceptualization of the study. Jo Erwin, Martha Paisi, Robert Witton, Sarah Neill and Lorna Burns were involved in the methodology of the study and also in conducting reviews in this study. Jo Erwin, Martha Paisi, Robert Witton, Sarah Neill, Tara Vassallo and Isaac Vassallo were involved in interpretation of the findings. Jo Erwin, Martha Paisi, Robert Witton, Sarah Neill, Lorna Burns, Urshla Devalia, Tara Vassallo, Isaac Vassallo, Abigail Nelder and Jo Erwin were involved in writing – original draft preparation. Jo Erwin, Martha Paisi, Robert Witton, Sarah Neill, Lorna Burns, Urshla Devalia, Tara Vassallo, Isaac Vassallo, Abigail Nelder and Jo Erwin were involved in writing – review and editing. All authors have read and agreed to the published version of the manuscript.

## CONFLICT OF INTEREST

The authors declare no conflict of interest.

## ETHICS STATEMENT

Ethical approval was not required for this review as data used for analysis were extracted from published studies.

## Data Availability

Data sharing is not applicable to this article as no new data were created or analysed in this study and no secondary data analysis was undertaken.

## APPENDIX 1 THE HUMAN DEVELOPMENT INDEX IS A SUMMARY MEASURE OF AVERAGE ACHIEVEMENT IN KEY DIMENSIONS OF HUMAN DEVELOPMENT: A LONG AND HEALTHY LIFE, BEING KNOWLEDGEABLE AND HAVE A DECENT STANDARD OF LIVING (WHERE 1.00 IS THE HIGHEST POSSIBLE VALUE AND 0.35 IS THE LOWEST POSSIBLE VALUE)

**Table:**

Countries with a very High Human Development Index	
Andorra	Australia
Argentina	Bahamas
Austria	Barbados
Bahrain	Belgium
Belarus	Bulgaria
Brunei Darussalam	Chile
Canada	Cyprus
Croatia	Denmark
Czechia	Finland
Estonia	Germany
France	Hong Kong, China (SAR)
Greece	Iceland
Hungary	Israel
Ireland	Japan
Italy	Korea (Republic of)
Kazakhstan	Latvia
Kuwait	Lithuania
Liechtenstein	Malaysia
Luxembourg	Montenegro
Malta	New Zealand
The Netherlands	Oman
Norway	Portugal
Poland	Romania
Qatar	Saudi Arabia
Russian Federation	Slovakia
Singapore	Spain
Slovenia	Switzerland
Sweden	United Kingdom
United Arab Emirates	Uruguay
United States	

## APPENDIX 2 SEARCH STRATEGIES

Search strategy for EMBASE

Embase <1974 to 2021 February 12>

1 exp Autism/74474

2 Autis*.ab,kw,ti.  68519

3 Pervasive development disorder*.ab,kw,ti. 141

4 Kanner*.ab,kw,ti. 332

5 (Asperg* not aspergill*).ab,kw,ti. 3761

6 Rett.ab,kw,ti. 4736

7 1 or 2 or 3 or 4 or 5 or 6 86514

8 dental health/4094

9 dental procedure/26330

10 exp tooth disease/220856

11 exp dentist/25749

12 (oral adj3 (health* or hygiene or care)).ab,kw,ti. 48814

13 dental.ab,kw,ti. 228647

14 ((tooth adj3 (health* or hygiene or care or brush* or floss*)) or toothbrush*).ab,kw,ti. 8182

15 (teeth adj3 (health* or hygiene or care or brush* or floss*)).ab,kw,ti. 4245

16 dentist*.ab,kw,ti. 74090

17 or/8‐16 439135

18 7 and 17 803

Ovid MEDLINE(R) and Epub Ahead of Print, In‐Process & Other Non‐Indexed Citations, Daily and Versions(R) <1946 to February 15, 2021>

**Table:**

1	exp Autism Spectrum Disorder/	31,735
2	Autis*.ab,kw,ti.	52,514
3	Pervasive developmental disorder*.ab,kw,ti.	2048
4	Kanner*.ab,kw,ti.	227
5	(Asperg* not aspergill*).ab,kw,ti.	2384
6	Rett.ab,kw,ti.	3632
7	1 or 2 or 3 or 4 or 5 or 6	59,204
8	Oral Health/	17,243
9	Oral Hygiene/	13,093
10	Dental Care/	21,609
11	dental health services/	4127
12	exp Tooth Diseases/	175,921
13	Dentists/	18,313
14	(oral adj3 (health* or hygiene or care)).ab,kw,ti.	44,013
15	dental.ab,kw,ti.	225,529
16	((tooth adj3 (health* or hygiene or care or brush* or floss*)) or toothbrush*).ab,kw,ti.	7995
17	(teeth adj3 (health* or hygiene or care or brush* or floss*)).ab,kw,ti.	3806
18	dentist*.ab,kw,ti.	77,372
19	8 or 9 or 10 or 11 or 12 or 13 or 14 or 15 or 16 or 17 or 18	414,449
20	7 and 19	494

Database—CINAHL Plus with Full Text

**Table:**

S1	((MH ‘Asperger Syndrome’) OR (MH ‘Autistic Disorder’) OR (MH ‘Pervasive Developmental Disorder‐Not Otherwise Specified’)) OR TI (Autis* or Asperg* or ‘pervasive developmental disorder’ or Rett or Kanner) OR AB (Autis* or Asperg* or ‘pervasive developmental disorder’ or Rett or Kanner) OR SU (Autis* or Asperg* or ‘pervasive developmental disorder’ or Rett or Kanner)	37,553
S2	(MH ‘Oral Health’)	12,752
S3	(MH ‘Oral Hygiene’)	5937
S4	(MH ‘Dental Care+’)	18,517
S5	(MH ‘Dental Health Services’)	1424
S6	(MH ‘Tooth Diseases+’)	35,558
S7	(MH ‘Dentists’)	10,488
S8	TI ((oral N3 (health* or hygiene or care))) OR AB ((oral N3 (health* or hygiene or care))) OR SU ((oral N3 (health* or hygiene or care)))	27,688
S9	TI dental or dentist* OR AB dental or dentist* OR SU dental or dentist*	136,992
S10	TI (((tooth N3 (health* or hygiene or care or brush* or floss*)) or toothbrush*)) OR AB (((tooth N3 (health* or hygiene or care or brush* or floss*)) or toothbrush*)) OR SU (((tooth N3 (health* or hygiene or care or brush* or floss*)) or toothbrush*))	5669
S11	TI ((teeth N3 (health* or hygiene or care or brush* or floss*))) OR AB ((teeth N3 (health* or hygiene or care or brush* or floss*))) OR SU ((teeth N3 (health* or hygiene or care or brush* or floss*)))	2398
S12	S2 OR S3 OR S4 OR S5 OR S6 OR S7 OR S8 OR S9 OR S10 OR S11	154,615
S13	S1 AND S12	377

Database—Dentistry & Oral Sciences Source

**Table:**

S1	DE ‘AUTISM’ OR DE ‘ASPERGER'S syndrome’ OR DE ‘AUTISM in adolescence’ OR DE ‘AUTISM in adults’ OR DE ‘AUTISM in children’	161
S2	DE ‘PERVASIVE child development disorders’	5
S3	TI (Autis* or Asperg* or ‘pervasive developmental disorder’ or Rett or Kanner) OR AB (Autis* or Asperg* or ‘pervasive developmental disorder’ or Rett or Kanner) OR SU (Autis* or Asperg* or ‘pervasive developmental disorder’ or Rett or Kanner)	487
S4	S1 OR S2 OR S3	489
S5	(DE ‘ORAL health’) OR (DE ‘ORAL hygiene’)	13,599
S6	(DE ‘DENTAL care’) OR (DE ‘DENTISTS’)) OR (DE ‘DENTAL pathology+’)	35,901
S7	TI ((oral N3 (health* or hygiene or care))) OR AB ((oral N3 (health* or hygiene or care))) OR SU ((oral N3 (health* or hygiene or care)))	33,270
S8	TI (dental or dentist*) OR AB (dental or dentist*) OR SU (dental or dentist*)	244,563
S9	TI (((tooth N3 (health* or hygiene or care or brush* or floss*)) or toothbrush*)) OR AB (((tooth N3 (health* or hygiene or care or brush* or floss*)) or toothbrush*)) OR SU (((tooth N3 (health* or hygiene or care or brush* or floss*)) or toothbrush*))	12,076
S10	TI ((teeth N3 (health* or hygiene or care or brush* or floss*))) OR AB ((teeth N3 (health* or hygiene or care or brush* or floss*))) OR SU ((teeth N3 (health* or hygiene or care or brush* or floss*)))	9454
S11	S5 OR S6 OR S7 OR S8 OR S9 OR S10	253,069
S12	S4 AND S11	306

Database – SocINDEX

**Table:**

S1	DE ‘AUTISM’ OR DE ‘AUTISM in adolescence’ OR DE ‘AUTISM in adults’ OR DE ‘AUTISM in children’	1981
S2	TI (Autis* or Asperg* or ‘pervasive developmental disorder’ or Rett or Kanner) OR AB (Autis* or Asperg* or ‘pervasive developmental disorder’ or Rett or Kanner) OR KW (Autis* or Asperg* or ‘pervasive developmental disorder’ or Rett or Kanner)	3827
S3	S1 OR S2	3909
S4	DE ‘DENTAL care’	624
S5	TI ((oral N3 (health* or hygiene or care))) OR AB ((oral N3 (health* or hygiene or care))) OR KW ((oral N3 (health* or hygiene or care)))	1063
S6	TI (dental or dentist*) OR AB (dental or dentist*) OR KW (dental or dentist*)	4373
S7	TI (((tooth N3 (health* or hygiene or care or brush* or floss*)) or toothbrush*)) OR AB (((tooth N3 (health* or hygiene or care or brush* or floss*)) or toothbrush*)) OR KW (((tooth N3 (health* or hygiene or care or brush* or floss*)) or toothbrush*))	208
S8	TI ((teeth N3 (health* or hygiene or care or brush* or floss*))) OR AB ((teeth N3 (health* or hygiene or care or brush* or floss*))) OR KW ((teeth N3 (health* or hygiene or care or brush* or floss*)))	148
S9	S4 OR S5 OR S6 OR S7 OR S8	4839
S10	S3 AND S9	4

Database—SCOPUS

(TITLE‐ABS‐KEY (autis* OR asperg* OR ‘pervasive developmental disorder’ OR rett OR kanner)) AND (TITLE‐ABS‐KEY (dental OR dentist* OR toothbrush*) OR TITLE‐ABS‐KEY (oral W/3 (health* OR hygiene OR care)) OR TITLE‐ABS‐KEY ((tooth OR teeth) W/3 (health* OR hygiene OR care OR brush* OR floss*))) AND NOT TITLE‐ABS‐KEY (aspergillosis) 855

Database—Web of Science

1. TOPIC: (autis* OR asperger* OR ‘pervasive developmental disorder’ OR rett OR kanner)

2. TOPIC: (dental OR dentist* OR toothbrush*) OR TOPIC: (oral near/3 (health* OR hygiene OR care)) OR TOPIC: ((tooth OR teeth) near/3 (health* OR hygiene OR care OR brush* OR floss*))

1 and 2

433

Database—Psychinfo

(MAINSUBJECT.EXACT.EXPLODE(‘Autism Spectrum Disorders’) OR ti(autis* OR asperger* OR ‘pervasive developmental disorder’ OR rett OR kanner) OR ab(autis* OR asperger* OR ‘pervasive developmental disorder’ OR rett OR kanner)) AND ((MAINSUBJECT.EXACT.EXPLODE(‘Oral Health’) OR MAINSUBJECT.EXACT.EXPLODE(‘Dental Treatment’) OR MAINSUBJECT.EXACT(‘Dental Health’) OR MAINSUBJECT.EXACT(‘Dentists’)) OR (ti(oral health OR oral hygiene OR oral care) OR ab(oral health OR oral hygiene OR oral care)) OR (ti(tooth health* OR tooth hygiene OR tooth care OR tooth brush* OR tooth floss* OR toothbrush* OR teeth health* OR teeth hygiene OR teeth care OR dental OR dentist*) OR ab(tooth health* OR tooth hygiene OR tooth care OR tooth brush* OR tooth floss* OR toothbrush* OR teeth health* OR teeth hygiene OR teeth care or dental OR dentist*))) 60

Grey literature searches

**Table:**

Organization	Web address	Search strings	Quick link to non‐Google search results
Public Health England	www.gov.uk	(‘oral health’ OR ‘oral hygiene’ OR ‘oral care’ OR dental OR dentist OR toothbrush OR toothbrushing OR teeth) AND (autism OR autistic OR asperger)	https://www.gov.uk/search/all?keywords=(%22oral%2Bhealth%22%2BOR%2B%22oral%2Bhygiene%22%2BOR%2B%22oral%2Bcare%22%2BOR%2Bdental%2BOR%2Bdentist%2BOR%2Btoothbrush%2BOR%2Btoothbrushing%2BOR%2Bteeth)%2BAND%2B(autism%2BOR%2Bautistic%2BOR%2Basperger)%2B%26order=relevance
Health Foundation	www.health.org.uk	via Google.co.uk: (‘oral health’ OR ‘oral hygiene’ OR ‘oral care’ OR dental OR dentist OR toothbrush OR toothbrushing OR teeth) AND (autism OR autistic OR asperger OR ‘pervasive developmental disorder’) AND site:www.health.org.uk	
National Institute for Health and Care Excellence	nice.org.uk	(‘oral health’ OR ‘oral hygiene’ OR ‘oral care’ OR dental OR dentist OR toothbrush OR toothbrushing OR teeth) AND (autism OR autistic OR asperger OR ‘pervasive developmental disorder’)	https://www.nice.org.uk/search?q=(%22oral%20health%22%20OR%20%22oral%20hygiene%22%20OR%20%22oral%20care%22%20OR%20dental%20OR%20dentist%20OR%20toothbrush%20OR%20toothbrushing%20OR%20teeth)%20AND%20(autism%20OR%20autistic%20OR%20asperger%20OR%20%22pervasive%20developmental%20disorder%22)
University of Cambridge Autism Research Centre	https://www.autismresearchcentre.com/	via Google.co.uk: (‘oral health’ OR ‘oral hygiene’ OR ‘oral care’ OR dental OR dentist OR toothbrush OR toothbrushing OR teeth) AND site:autismresearchcentre.com	
National Autistic Society	https://www.autism.org.uk	via Google.co.uk: (‘oral health’ OR ‘oral hygiene’ OR ‘oral care’ OR dental OR dentist OR toothbrush OR toothbrushing OR teeth) AND site:autism.org.uk	
Scottish Autism	https://www.scottishautism.org/	via Google.co.uk: (‘oral health’ OR ‘oral hygiene’ OR ‘oral care’ OR dental OR dentist OR toothbrush OR toothbrushing OR teeth) AND site:scottishautism.org	
Autistica	https://www.autistica.org.uk/	via Google.co.uk: (‘oral health’ OR ‘oral hygiene’ OR ‘oral care’ OR dental OR dentist OR toothbrush OR toothbrushing OR teeth) AND site:autistica.org.uk	
British Society for Disability & Oral Health	https://bsdh.org	via Google.co.uk: (autism OR autistic OR asperger OR ‘pervasive developmental disorder’) AND site:https://bsdh.org	
British Society for Paediatric Dentistry	https://www.bspd.co.uk	via Google.co.uk: (autism OR autistic OR asperger OR ‘pervasive developmental disorder’) AND site:https://www.bspd.co.uk	
British Dental Association	https://bda.org	via Google.co.uk: (autism OR autistic OR asperger OR ‘pervasive developmental disorder’) AND site:https://bda.org	

## APPENDIX 3 STUDY CHARACTERISTICS

**Table:**

Author's surname and year of publ.	Aim of study	Country, city/region	Type and design of study	Intervention	Recruitment, data collection	Participant population, sample size and characteristics	Results relating to: Oral health behaviours, Access to dental care, Provision of dental care as given by authors	Data qual score
Alhammad et al. (2020)	To assess the knowledge, attitude, and practice regarding oral health care among parents of autistic children and the challenges faced in accessing dental care for them.	Saudi Arabia	Quantitative descriptive		2 Autism societies; questionnaire	Parents of children with an autism spectrum disorder (ASD) diagnosis (*n *= 263).	ACCESS: 54% report difficulty in locating an appropriate dental clinic for their ASD child; 50.6% report difficulty in managing their child while waiting in the dental clinic waiting room; 32.7% report that dentists refused to care for their children; and 49.4% report that medical insurance does not cover their ASD dental cost needs	60
			Cross‐sectional					
Alhumaid et al. (2020)	To assess the association between oral health status and oral health practices of children with ASD in relation to their parental attitudes and comfort in providing oral care.	Saudi Arabia, Riyadh	Quantitative descriptive		13 Special needs schools, questionnaire.	Parents of children with ASD attending special needs schools *N *= 75; age of children 6–18 years. 66% Male. Mean age ± SD = 10.8 ± 3.1 years.	ORAL HEALTH: Association between positive parental attitudes towards oral health and sugar consumption—their children consumed significantly less sugar (*p *= .043); Oral health practices not associated with parental attitudes or level of comfort in providing care.	60
			Cross‐sectional					

Alshihri et al. (2020) (1)	Investigate parental views and experiences regarding dental care for their ASD children; analyse barriers and facilitators influencing ASD children's access to dental care services.	Saudi Arabia, Riyadh	Quantitative descriptive		2 Autism societies, questionnaire.	Mothers of children with ASD (*n *= 142). Age 22–57 years, mean 38.0. Educational level: Primary 2.8%, intermediate 12%, high school 28.2% and university 57%. Economic level: Low 12.7%, middle 83.1% and high 4.2%.	ACCESS: 68.3% perceived difficulties in finding dental care. Barriers included cost of treatment (75.4%), finding a dentist to treat the ASD child (74.6%) and behaviour of their ASD child (45.1%). Noise at dental clinics was the most frequently reported trigger for their ASD child (64.9%). 86% of mothers whose child had been to a dentist reported that the uncooperative behaviour of their child was the biggest problem when attending a dentist's appointment. 37% reported that ‘most dentists don't know how to deal with ASD children’. Medical insurance and previous bad experience had a significant impact on difficulty in finding dental care (*p* < .05).	40
			Cross‐sectional					
Alshihri et al. (2020) (2)	To investigate parental challenges regarding home oral health care of their autistic children and experience of supervising teeth brushing.	Saudi Arabia, Riyadh	Quantitative descriptive Cross‐sectional		2 Autism societies; questionnaire.	Mothers of children with ASD (*n* = 142). Age 22–57 years, mean 38.0. Educational level: Primary 2.8%, intermediate 12%, high school 28.2% and university 57%. Economic level: Low 12.7%, middle 83.1% and high 4.2%.	ORAL HEALTH: Significant positive association between brushing frequency and allowing brushing supervision (*p* < .001). No significant association between children's age, gender or autism severity and allowing brushing supervision.	60

Backman et al. (1999)	To present and evaluate a model based on visual pedagogics to introduce dentistry to preschool children with autism.	Sweden, Vasterbotten	Non‐randomized Analytical cross‐sectional	TEACHH model using social story boards at home and at the dentist to prepare the child for a dental visit; agreed consistent dentist‐related vocabulary.	Public Dental Health clinic—clients of Team Autism; members of the National Autism Society telephone interviews and data review.	Children: Cases (*n *= 16), Controls (*n *= 16). Aged 3–6 years (mean: 2.8 years). Cases 94% male; controls' gender mix not given.	ORAL HEALTH: Postintervention in cases % of children brushing 1 or 2× per day with FL toothpaste increased from 75% to 100%; No change in % of children eating sugary snacks.	20
							ACCESS: Parents reported that ‘the most important factors for them’ was the competence of the dentist, dentist's knowledge of autism, the clinic is calm and appointments are on time (to reduce waiting times).	
Barry, (2014)	To examine the problems encountered by children with ASD when accessing dental care.	UK, Hull & East Riding	Nonrandomized study		Dental clinic/special schools, survey.	Cases (*n *= 56): Parents/carers of children with ASD. Controls (*n *= 56): Parents/carers of age‐matched healthy, neurotypical children. Matched by age and postcode.	ACCESS: No significant difference in accessing dental care between the study and control groups, but access was perceived as more difficult in the ASD group (*p *< .001). Significantly greater perceived difficulty in travelling to the dental surgery and more frequent predictions of negative behaviours in the ASD group.	40
			Cross‐sectional case‐control					

Brickhouse et al. (2009)	To examine the reported use of dental services for families of children with ASD and identify barriers that affect their access to dental care.	USA, Virginia	Quantitative descriptive		Caregivers registered with the Autism Programme of Virginia; questionnaire.	Caregivers with at least one child with ASD (*n *= 55). High school diploma 34%, Some college 31%, Associate's degree 31% and Bachelor's degree 4%. Household income < $20,000 8%, $20,000–$49,999 33% and $50,000 59%.	ACCESS: Most cited reasons why: not scheduled for checkup in the next 12 months: cannot find a dentist who treats children with special needs 16%; no services available 11%; difficulty in locating a dentist: very difficult or cannot locate 15%; somewhat difficult 37%; and somewhat easy to easy 48%. Ever refused treatment 25%. History of child's behaviour in dental office: (%) Cooperative 41, somewhat uncooperative 26 and extremely uncooperative 33. Respondent's education, child's age, race and history of behaviour in the dental office significantly related to ‘time since last dental visit’. Respondents' education, income and insurance coverage and child's history of behaviour in the dental office significantly related to having a dentist for periodic dental care. Insurance coverage, household income and child's age and history of behaviour in the dental office significantly related to whether a child was ‘unable to receive dental care when needed in the last year when needed’. Respondent's household income and child's history of difficult behaviour in the dental office significantly related to ability to receive care when needed/has regular dental provider. Multivariate regression—the child's history of behaviour in the dental office and household income were significant for dentist for periodic oral health care (P < .001 and P < 01, respectively), those with ‘extremely uncooperative’ behaviour and income $20,000—$49,000 less likely to have a regular dentist. These factors were significant for ‘unable to get care in the last year when needed’ (P < 001 and *p* < .02 respectively).	20
			Cross‐sectional					
Brown et al. (2014)	To identify what parents perceive to be good practice in the care of children with autism in dental settings and to highlight difficulties in accessing dental services.	UK, Lancashire	Mixed methods Pilot cross‐sectional		Parents who attended autism conference; questionnaire interviews.	Parents of children with ASD (*n *= 19).	ACCESS: 68.4% reported no barriers accessing dental services and 31.5% reported experiencing barriers to accessing dental services. 63% reported experiencing difficulties related to their ASD when accessing dental services. Parents reported negative experiences often caused by communication breakdown between the child, parent and dental staff; lack of practitioners' awareness of ASD; reluctance to adapt practice to cater for sensory difficulties. Parents who reported positive experiences felt they were listened to by dental staff and that practice was altered to meet individual needs. Extra time to explain process and procedures worked well.	60
Cagetti et al. (2015)	To present a multistage approach based on the use of visual supports to facilitate children with ASDs to undergo an oral examination and treatments.	Italy, Milan	Nonrandomized Experimental before and after study.	Visual training to undergo four stages: An oral examination (Stage 1), a professional oral hygiene session (Stage 2), sealants (Stage 3) and, if necessary, a restorative treatment (Stage 4).	Hospital Childhood Neuropsychiatric clinic, Autism and PDD Diagnosis and Care centre; scales, observation.	Child patients (*n *= 83). Six to Twelve years, 63.1% male. Intellectual disability—None = 24%, mild = 25.3%, severe = 50.6%. Verbal fluency—Fluent = 8.5%, nonfluent = 38.5%, nonverbal = 22.8%.	PROVISION: 92.8% of subjects accepted oral exam and professional tooth cleaning; 84% accepted sealant. Of the 44 subjects who needed restorative treatments (Stage 4), 93% accepted. Gender and age group were not statistically associated with the acceptance rate at any stage. The acceptance rate at each stage was statistically significantly associated with verbal fluency (*p *= .02; *p* =.04; *p* = .01, respectively, for Stages 1, 3, and 4). The verbal/intellectual/cooperation dummy variable was statistically associated with the acceptance rate (*p *< .01).	20

Cermak et al. (2015)	Pilot and feasibility study to examine the impact of a sensory adapted dental environment (SADE) to reduce distress, sensory discomfort and perception of pain during oral prophylaxis for children with ASD.	USA,		In the control condition (RDE), dental cleaning administered in a standard manner. In the experimental condition (SADE), the same room and dental practitioners were utilized, but the SADE procedures were enacted to modify the sensory input that the child experienced.	Urban children's hospital; questionnaire and physiological tests and records.	Children (*n *= 44—22 = ASD, 22 = neurotypical). Age 6–12 years. Mean age ASD 8.2 typical development (TD) 8.3; anxiety scale ASD 9.1 TD 5.9 (signif); gender ASD male 81.8% TD male 45.5%; race ASD Caucasian 95.5% TD 81.8%; ethnicity Hispanic ASD 81.8% TD 68.2%. Maternal education level: High school or less ASD 18.2%, TD 40.9%; vocational/college ASD 68.2% TD 27.3%; degree%2B ASD 13.6% TD 31.8%. Paternal education level: High school or less ASD 52.4%, TD 33.3%; vocational/college ASD 23.8% TD 33.3%; degree%2B ASD 23.8% TD 33.3%.	PROVISION: Both the ASD and neurotypical groups showed decreased physiological anxiety (as measured by EDA). Child report measures of pain intensity and sensory discomfort were significantly improved in the SADE environment for both groups. Effect sizes were moderate to large in the ASD group and small to moderate for the neurotypical group. There was an increase in the proportion rated as relaxed and co‐operative in the ASD group (46% RDE 59% SADE *p*= .08). When the Frankl scale results were stratified into negative and positive categories, there was an increase in positive behaviour rating in the ASD group from 54% RDE to 64% SADE (cf neurotypical group 91% RDE vs. 95% SADE). Cost savings—duration of cleaning was significantly longer in the SADE condition, but the number of hands required for restraint was significantly reduced in the SADE condition.	40
			Nonrandomized crossover design					
Du et al. (2015)	To determine the associations between autism developmental profiles and cooperation with an oral health screening among preschool children with ASD.	Hong Kong	Nonrandomized study		Special Child Care Centres; assessment and oral exam.	Children (*n* = 347). Aged 25–77 months. Mean age 57.5 (±11.7 months); male 85%.	PROVISION: In regression analyses accounting for age and gender, inability to cooperate with an oral health screening was associated with high level of challenging behaviours (OR: 10.50, 95% CI: 2.89–38.08, *p*\0.001) and reduced cognitive functioning (OR: 5.29, 95% CI: 1.14–24.61, *p* = .034). Feasibility of conducting oral health screening in preschool children with ASDs was associated with their cognitive functioning (*p* = .001), social skills development (*p* = .002), communication skills development (*p*\0.001), reading skills (*p*\0.001) and challenging behaviours (*p* = .06). Age was positively associated with likelihood of cooperative behaviour with an oral health screening (OR: 1.06, 95% CI: 1.03, 1.08, *p*\0.001).	100
			Cross‐sectional					
Du et al. (2019)	To compare oral health behaviours and barriers to dental care among preschool children with and without ASD; to evaluate the dental knowledge and attitudes of their parents.	Hong Kong	Nonrandomized study		Cases (CS): Special Childcare Centres.	Parents of children with ASD (*n *= 257) and parents of non‐ASD children (*n *= 257). Age, gender and ethnicity not reported. Parents education level: None/primary ASD 6.2%, non‐ASD 8.4% (*p *= .004); secondary school ASD 47.3%, non‐ASD 59.8%; tertiary ASD 46.5%, non‐ASD 31.7%. Family income (HKD per month): <$10,000 ASD 13.6% non‐ASD 19.2% (*p *< .001); $10,000–$19,999 ASD 31.1%; non‐ASD 48.5%; $20,000%2B ASD 55.3% non‐ASD 32.6%.	ORAL HEALTH: Barriers to oral self‐care: 46.1% CS 19.7% CT reported that toothbrushing is a difficult task (*p *< .001). Reasons for difficulties: scared of toothbrushing—36.9% CS 13.6% CT (*p *= .0004); does not understand toothbrushing 46.8% CS 25.0% CT (*p *= .013); does not like anything in his/her mouth 36% CS 13.6% CT (*p *= .006).	100
			Cross‐sectional					
					Controls (CT): Mainstream preschools; questionnaire.			
							ACCESS: Significant difference in reported barriers to access to dental services among preschool children with and without ASD (*p* < .001). Inability to find a dentist willing to treat the child (*p* < .001); finding a dentist near the child's home (*p* < .001); dental staff being anxious or nervous about treating the child (*p* < .01); time to take child to the dentist (*p* < .05) and dental costs (*p* < .001); child does not like to have anything done in his/her mouth (*p* < .001); child cannot behave cooperatively at the dentist's clinic (*p* < .001); child's medical conditions make dental treatment very complicated (*p* < .001); and child has other more urgent health care needs (*p* < .001).	
Eades (2019)	To investigate UK dental professionals' knowledge of autism, their perceived confidence when treating autistic patients and the factors that affect this.	UK (national)	Mixed methods		NHS dentists directory; on‐line survey.	Dental health professionals (*n *= 357): Mean age: 44.60 (range: 22–70); 26.3% male; 84.0% White.18.8% GDP; 16.2% paediatric dentist; 8.4% special care dentist; 10.4% dental nurse; 4.5% hygienist; 36.7 orthodontist; 3.9% therapist; 2% community dentist; 11.8% other. Mean years in practice 20.8 (range: <1–25); Sector work—50.0% NHS; 12.2% Private practice, 37.8% Mixed; practice—36.1% General practice, 19.0% Community practice; 29.4 Hospital, 15.5% Other. 38% reported having had personal experience of autism.	PROVISION: 65.9% reported autistic patients more difficult to treat. Of those with experience of treating autistic patients, 40.9% reported being unaware of useful techniques and resources for treating autistic patients. 24.6% did not use any special techniques or resources. 44.7% had received training in autism. Respondents were ‘somewhat confident’ in ability to treat autism patients. Least confident in knowledge of relevant local care pathways/services for people with autism. GDPs significantly less confident than paediatric dentists and special care dentists (*p *< .001). Self‐efficacy positively correlated to number of autistic patients treated each month (*p *< .001). Self‐efficacy scores higher in those who had undertaken autism training and who had personal experience of autism.	40
			Cross‐sectional					
Fakhruddin et al. (2017)	To evaluate the effectiveness of audiovisual (AV) distraction in behaviour modification during dental caries assessment and sealant placement in children with ASD.	United Arab Emirates, Sharjah	Nonrandomized Observational before and after study.	One introductory (desensitization) appointment and three treatment sessions. During treatment sessions, watched a movie projected on a screen with or without video eyewear.	Special Needs Teaching Clinic at University Dental Hospital; questionnaire, physiological tests.	Children (*n *= 28). Age 6.5–9.8 years. Mean age 7.5 years. 60.7% male. ‘Most diagnosed with moderate to mildly severe autism’.	ACCESS: A significant difference (*p* < .02) was observed in the mean heart rate during dental screening of the upper and lower jaws with and without video eyewear. A decrease was observed in the mean heart rate during subsequent treatment sessions.	20

Fenning et al. (2020)	To test the hypothesis that (1) children with ASD participating in the Autism Treatment Network (ATN) would not differ from NSCH children without special health care needs in reported preventive dental services; (2) that rates of preventive dental care for ATN children, the majority of whom have comorbid intellectual disability, would be similar to NSCH children with parent‐identified ASD among whom intellectual disability is less prevalent.	USA, National	Nonrandomized study		Families enroled in Autism Speaks ATN (*n *= 375 families); questionnaires, National Survey of Children's Health data, testing.	Parents and children. Children ATN RCBA (*n *= 375): Age: 4–17 years, mean age: 9.7; male: 81.1%; race: 82.2% White; 4.7% Asian; 5.2% African American/Black Canadian; other 7.9%. Ethnicity 93.1% non‐Hispanic; 6.9% Hispanic. Mean IQ: 76.1 (SD: 23.0). Mean adaptive behaviour: 71.4 (SD: 15.4). Mean ASD symptom severity 105.5 (SD: 28.1). Mean behaviour problems 60.9 (SD: 9.2). Highest education level of caregivers: Less than degree 33.3%; degree or above 66.7%. Insurance type: None 3.7%; public (30.9%); private (49.9%); public and private (15.5%). Annual family income ≤ $24,999 44 (14.5%); $25,000–$49, 999 73 (24.1%); $50,000–$74,999 59 (19.5%); $75,000–$99,999 39 (12.9%); ≥$100,000 88 (29.0%).	ACCESS: Families with only private insurance received significantly more preventive dental services and increased likelihood of reported dental visit success (average 4.3 per person) as did participants with a parent/carer with degree education (4.2). In multivariate analysis, higher level of child IQ and fewer parent‐reported behaviour problems were associated with greater likelihood of dental visit success. Higher level of child adaptive behaviour and IQ were significantly associated with receiving a greater number of preventive dental services.	80
			Cross‐sectional					
Hauschild et al. (2019)	To investigate parents'/caregiver's experiences and perceptions when attempting to seek or receive oral health care for their child with autism.	USA, Massachusetts	Qualitative		Caregivers of children attending three autism centres; focus groups.	Parent/caregiver (*n *= 17). Mean age: 42; 12% male; 88% Caucasian, 6% Hispanic, 6% Asian. Education: Never finished 6%, high school 6%; some college 23.5%, associate degree 17.5%, batchelor degree and above 47%. Marital status: Single 17.5%, married 76%, divorced 6.5%. Insurance: Private 35.3%, state funded 35.3%, both 29.4%.	ACCESS: Themes: barriers to specialized dental care; lack of provider ASD knowledge and clinical experience; and professional compassion and ASD sensitivity.	100
			Exploratory qualitative descriptive					
Holt (2018)	To evaluate a real‐time text messaging service (RTMS) to improve dental attendance experience for CYP with ASC.	UK	Nonrandomized study.	Parent/carer texts RTMS to confirm arrival at clinic site; RTMS confirms receipt of text. When dentist ready, the RTMS texts parent/carer. Parent/carer and patient go straight into dental appointment.	Special Care Dental Service; questionnaire	Parents *N *= 16, carer *N *= 1. No demographic information given. No information given.	PROVISION: 93% agreed or strongly agreed RTMS service useful; 87% agreed or strongly agreed RTMS reduced the amount of time they had to wait compared to previous appointments; and 87% agreed or strongly agreed RTMS helped their child. Parents commented that RTMS reduced stress and anxiety in both parents and CYP.	40
			Cross‐sectional service evaluation.					
Isong et al. (2014)	To determine if an innovative strategy using two types of electronic screen media was feasible and beneficial in reducing fear and uncooperative behaviours in children with ASD undergoing dental visits.	USA, Boston	RCT	Subjects randomly assigned to 1 of 4 groups: (A) control (usual care); (B) video peer modelling of dental visit; (C) video watching a movie during the dental visit using video goggles; and (D) video peer modelling plus video goggles.	Hospital Dental Clinic; parental reports, rating scales, physiological tests.	Children *N *= 80. Aged 7–17 years. 81% males, with an average age of 9.9 years (SD: 2.44). 53% Caucasian, 16% African American, 9% Asian, 4% Hispanic and 16% other. 60% of subjects' parents had at least a college degree.	PROVISION: Between Visits 1 and 2, the mean anxiety and behaviour scores decreased significantly by 0.8 points (*p* = .03) for subjects with video goggles only and video peer modelling plus video goggles. No significant changes within control and video peer modelling only. No significant difference in the mean anxiety and behaviour scores between groups over time.	40
			Pilot RCT					
Lai et al. (2012)	(a) To describe the unmet dental needs and associated barriers to oral health care among children with ASD. (b) To examine the association of having unmet dental needs with (1) the type of ASD and (2) the child's perceived behaviour in the dental office.	USA, North Carolina	Quantitative descriptive		Members Autism Registry of North Carolina; questionnaire.	Caregivers (*n *= 555). No information on age or gender. Average household income <$35,000 26.6%, $35,000–74,999 36.7%, >$75,000 36.7%. Caregiver's education: Did not complete HS 3.3%, HS graduate 40.1%, College graduate 56.6%. Type of community: Rural 33.6%, suburban 50.7%, urban/city 15.7%.	ACCESS: 11.7% reported child needed dental care, but did not get it in the past 6 months. Main problems at last dental visit (*N* = 523): Child could not cooperate 29.8%, clinic did not accept Medicaid 21.0%, dentist/assistant not able to handle child 9.6% and dentist did not treat special needs children 8.2%. Most reported barriers to dental care for those who could not receive care when needed in the past 6 months (*N* = 65): Child uncooperative 60.0%, could not afford it 38.5% and No insurance 23.1%. In multivariate analysis, children with fair/good behaviour had decreased odds of having unmet dental needs (OR = 0.41, 95% CI = 0.19–0.86, *p* = .01; OR = 0.37, 95% CI = 0.17–0.80, *p* = .01) compared to those with poor behaviour.	60
			Cross‐sectional survey					
Lefer et al. (2018)	To present a training programme for teaching children and adolescents with ASD to be compliant with a dental examination.	France, Nantes	Nonrandomized	Dental examinations were performed in education centres by a paediatric dentist using a visual activity schedule on an iPAD, using the digital application cATED. cATED is a digital diary in using personalized pictograms, photos and schedules can be personalized.	School/care centre; observation and recording.	Children and adolescents (*n *= 52). Age: 3–19 years. Mean age 10.2 years. Male 86.5%.	PROVISION: 25% of participants underwent the entire dental exam at the beginning of the study—increased to 65.4% after 8 months. 7.7% not anxious at the beginning of the study—increased to 59.6% after 8 months.	20
			Experimental before and after study.					
Lewis et al. (2015)	To better understand problems in dental and oral care encountered by children with ASD.	USA, Seattle	Qualitative		Children's Autism Centre; focus groups.	Parents (*n *= 20). Male 40%.	ACCESS: Themes—(1) There is variability between children with ASD in how they tolerate dental and oral care and in what facilitates such care. (2) Parents want more extensive dental care for their children with ASD. (3) Each child's dental and oral care should be individualized based on parents' input about the unique characteristics and needs of their child.	80
			Grounded theory					
Logrieco et al. (2020)	To provide an understanding of the challenges experienced by children with ASD, their families and dentists during oral care treatment.	Italy, Abruzzo	Quantitative descriptive		Parents of children attending local nurseries, schools, rehabilitation centres and social associations. Dentists in the targeted area; questionnaires.	Parents of ASD (*n *= 57) and TD (*n *= 275) children. Mean age in years TD 42.7, ASD 40.6 dentists *n *= 61. Mean age 40.2. 68% male. Dentists: 10% paediatric dentists. 63.9% have at least one patient with ASD.	ORAL HEALTH: Statistically significant parent‐reported differences between TD and ASD children % TD/ASD—Never follow home dental prevention rules 10.5/18.9.	40
			Cross‐sectional					
							ACCESS: Statistically significant parent‐reported differences between TD and ASD children (%) TD/ASD—aggressive behaviour during the first visit 14.5/39.6. Rejection of treatment by dentists 1.3/12.1. Respondents consider dental care highly important 83.8/67.2.	
							PROVISION: 30% of dentists who treated children with ASD had attended a specialist course on the topic.	
Mah et al. (2016)	To test whether a visual schedule system using picture communication symbols can help children with autism have successful routine dental cleaning visits.	Canada, Vancouver	RCT	Patients randomly assigned to a control group, who received the tell‐show‐do method (standard of care), or a test group, who received a tell‐show‐do method plus a visual schedule system—a pictorial cue board to show the steps in a dental visit—created based on the Picture Exchange Communication System.	Dental clinic in children's hospital; observation and recording, completion of scales and measure of EDA.	Children (*n *= 14) 7 cases and 7 controls. All male. Cases mean age in months 72.86 (SD: 11.95), range: 39–81; Controls' mean age in months 55.86 (SD: 13.40), range 55–89. (*p *< .05). Average severity of autism symptoms in both groups = mild.	PROVISION: Patients in test group completed an average of 1.38 more steps, at 35.52 s per step faster, and with 18.7% lower levels of behavioural distress than those in the control group. Overall, across appointments, cases reported lower levels of behavioural stress compared to the control group. The greatest magnitude of benefit using visual pedagogy was in the first appointment.	60
			Pilot RCT					
Mansoor et al. (2018)	To investigate the challenges faced by ASD children and their families in Dubai from three different perspectives of dental care: Oral care at home, oral care at the dentist and access to oral care and to compare the results to their normally developing peers.	Dubai	Nonrandomized study		Cases (CS): 5 special needs centres.	Parents/guardians of autistic children (*n *= 151) and nonautistic children (211).	ORAL HEALTH: Child resists having teeth cleaned at home: Always CS 24.4%, CT 0.0% *p*< .001. Occasionally/rarely CS 75.6% CT 1000%. Child dislikes feeling of toothbrush in the mouth: Yes CS 53.0%, CT 5.7% *p*< .001.	40
					Controls (CT): Mainstream schools; questionnaire.			
			Case‐control					
							ACCESS: Child's experience at last dental visits rated as negative: CS 37%, CT 9.5% (*p *= .006). Children's uncooperative behaviour increased at the dentist's clinic: CS 66.6%, CT 16.7% (*p *< .001). Children's sensory sensitivities increased at the dentist's clinic: CS 56.4%, CT 7.5% (*p *< .001). If the child had to go to the dentist tomorrow, he or she would be extremely afraid: CS 32.7%, CT 7.5% (*p *= .009). Child's reaction to the dentist will not encourage them to take him/her for regular dental checkups: CS 38.2%, CT 16.7% (*p *= .023). % of children that fear, dislike and complain of: Dentist drill CS 46.3, CT 41.5; bright light CS 27.8 CT 2.4 (*p *= .001); Loud sound CS 31.5 CT 12.2; Leaning back in dentist chair CS 46.3 CT 7.3.	
Marshall et al. (2007)	To evaluate potential predictors of cooperation during dental appointments for children with autism.	USA, Seattle	Quantitative descriptive		Childrens' Hospital Dental Clinic, University Childrens' Dental Clinic, private dental clinics; questionnaire, direct questioning of parents by members of dental team, dentist treatment notes.	Parent and child pair (*n *= 108). Children age: range: 2.7–19 years, mean age: 9.8 years. Gender: Male: 74%. Ethnicity: Caucasian 73%, Asian 17%, Black 8%, Native American 2%. Insurance: Medicaid 58%, dental insurance 39%, self‐pay 3%. Child lives at: Home 91%, foster care 4%, special school 4%, care facility 1%.	ACCESS: Trend for younger children to be less cooperative than older children (*p *= .06). Appointment type significantly predictive of behaviour—Emergency care 100% uncooperative, 68%, 62% and 33% uncooperative for initial exam, recall exam & operative care, respectively. Potential ‘risk factors’ for uncooperative behaviour: age (4–7 vs. 7); reading (no vs. yes); toilet training (no vs. yes); concurrent diagnoses (yes vs. no); and expressive language (no vs. yes). Having 2%2B ‘risk factors’ strongly significantly associated with uncooperative behaviour (*p *< .001). Child participation with toothbrushing was significantly predictive of cooperation (*p *= .004).	60
			Cross‐sectional					
McKinney et al. (2014)	To test hypotheses that lacking a medical home or having characteristics of more severe ASD is positively associated with having unmet dental need among children with ASD.	USA, National	Quantitative descriptive		2009–2010 National Survey of Children with Special Health Care Needs.	Parents (*n *= 2772). Household federal poverty level (FPL)1: ≤100% FPL 13.9%, 101%–200% FPL 18.9%, 201%–400% FPL 30.2%, >400% FPL 27.9% Primary language in home: English 96.5%, other 3.5%. Insurance type: Private 50.6%, public 27.3%, private and public 15.8%, uninsured 2.7%.	ACCESS: 15.1% of children with ASD (*n *= 376) had unmet dental need. Strongest predictor of unmet need was having a medical home or not (aOR: 2.61, 95% CI: 1.60, 4.24). Children with ASD with intellectual disability or greater communication or behavioural difficulties had greater odds of unmet dental need. ASD severity not associated with unmet dental need. Autistic children with more perceived behavioural difficulties had increased odds of unmet dental need (aOR: 3.35, 95% CI 1.69, 6.67). Children with ASD and more communication (or physical function) difficulties had higher unmet dental need (*p *= .05). Children whose ASD interfered with their ability to attend school and organized activities were at greater odds of unmet dental need compared to those whose ASD did not interfere with these activities (aOR: 4.36, 95% CI: 2.16, 8.78). For children with unmet dental need, top parent‐reported barriers to dental care were cost, condition/behavioural characteristics of child, insurance and difficulty finding a dental provider.	100
			Cross‐sectional					
Murshid et al. (2017)	To evaluate the effectiveness of a specially designed dental book (preparatory aid) on the behaviour of a group of ASD Saudi children during their first dental visit.	Saudi Arabia, Riyadh	Nonrandomized study Cross sectional ‘Double‐blinded pre/postclinical study’.	Children's book to assist children and parents in preparing for the first dental visit. The book describes dental clinic, staff and procedures. Parents asked to read book with child every day at the same time as the future dental appointment. On the day of the appointment, children received oral exam, prophylaxis and topical FL application by dental students.	University's College of Dentistry; parent questionnaire, clinical oral examination of child, child behavioural evaluation.	Parent/child pair. Children—*N *= 40. Male 75%; age: 5–9 years; average age: 6.1 years. Children's disability: Mental disabilities and seizures 20%, no disability 80%. PARENTS: Mothers' educational level: Elementary 17.5%, secondary 5.0%, high school 37.5%, diploma 5.0%, college 35.0%. Fathers' educational level: Elementary 7.5%, secondary 2.5%, high school 27.5%, diploma 7.5%, college 55.0%. Family income (Saudi Riyals): <5000 5.0%, >5000–10,000 50.0%, >1000–20,000 37.5%, >20,000 7.5%.	ACCESS: As reported by parents—effect of using aid on the child's behaviour: 27.5% absolutely not effective, 20.0% not effective, 15.0% neutral, 17.5% less effective, 17.5% effective and 2.5% highly effective. Effect of using aid on parents' dental knowledge: 2.5% not effective, 30.0% effective and 67.5% highly effective. Effectiveness after 4 months of follow‐up—57.5% used irregular toothbrushing at baseline, this reduced to 35%, and one‐time toothbrushing increased from 17.5% to 42.5% (*p *= .021). Positive behaviour during treatment increased from 47.5% to 80% (*p *< .001). Dental problems reduced from 50% to 30% (*p *= .005).	20
Narzisi et al. (2020)	To describe an experience of dental care supported by Information and Communication Technologies (ICT) for children with ASD in a public health service.	Italy, Pisa	Nonrandomized Before and after feasibility study.	MyDentist—an intervention combining ICT behavioural techniques and a specific clinical protocol designed for autistic children.	Hospital Paediatric Dental Clinic; questionnaire.	Parent/child pair. Children *N *= 59. Gender: Male 76.3%. Age: range: 4–16, mean age: 9.9 years (SD: 5.43). DSM‐5 ASD severity: Level 1: 8.5%, Level 2: 62.7%, Level 3: 28.8%. Cognitive functioning: Normal range: 8.5%, mild: 6.8%, moderate: 47.4%, severe: 32.2%. Language: No words: 49.1%, 2–3 words: 23.7%, fluent: 27.1%.	ORAL HEALTH: Statistically significant increase in parent‐reported oral health behaviours before and after the intervention: Child able to follow rules for proper dental hygiene, child capable of using toothbrush, child capable of putting toothpaste on toothbrush, child brushes teeth independently, child brushing teeth after meals.	
							ACCESS: Statistically significant increase in parent‐reported behaviours: Child becomes upset when he has to go the dentist.	
Nelson et al. (2017)	To evaluate the effectiveness of a dental desensitization programme for children with ASD and determine the characteristics associated with a successful dental examination.	USA, Seattle	Nonrandomized study	Desensitization programme with an individualized care plan including goal setting and previsit preparation at home and use of social stories. At each visit, tailored BGTs such as voice control and individualized positive reinforcement were used.	Dental desensitization programme at University Centre for Paediatric Dentistry; previsit information intake form, chart abstraction of child's clinical visit, behavioural rating score by dentist.	Parent/children (*n *= 168). Gender: Male = 82.7%, age: range: 4–18. Race: White: 50.6%, Asian: 9.5%, Black or African American: 11.3%, Other or multiple: 16.7%, Unanswered: 11.9%. Insurance: Public: 51.8%, Private: 47.0%, none: 1.2%.	PROVISION: An MTE was achieved for 77.4% of all children within 1–2 visits and 87.5% in five visits or less. Several factors predicted a successful dental examination: Ability to be involved in group activities (relative risk [RR], 1.18; *P* ¼ .02), ability to communicate verbally (RR, 1.17; *p* < .01), understanding of most language (RR, 1.14; *P* ¼ .02), moderate versus severe caregiver‐rated ASD severity (RR, 1.24; *P* ¼ .04) and ability to dress self (RR, 1.27; *P* ¼ .04).	40
			Retrospective cohort study					
Orellana et al. (2019)	To assess the effectiveness of a psychoeducational intervention programme designed to facilitate the performance of a series of steps of oral examination in children, adolescents and adults with autism spectrum disorder (ASD).	Chile, Bio Bio region	Nonrandomized study	Intervention included five sessions: (1) Familiarization, T‐S‐F and visual pedagogy. (2) Audiovisual modelling to demonstrate 10 steps of the oral examination. (3) In vivo modelling of steps. (4) Behavioural training in each of the 10 steps. (5) Self‐modelling—observing their own performance in previous photographed session.	Educational centres and organizations for people with ASD; questionnaire completed by social worker.	Children (*n *= 52), adolescents (*n* = 22). (%) Age: mean 6.13/12. Range: 4–9/10–17. Gender: Male: 88.5/77.3, insurance: Public: 69.2/86.4, private: 23.1/4.5, others: 7.7/9.1. Maternal education level: Primary: 0/4.5, secondary: 28.8/63.6, higher: 71.2, 31.8. Paternal education level: Primary 5.8/13.6, secondary: 28.8/22.7, higher: 65.4, 45.5, unknown: 0/18.2.	PROVISION: 74 children and adolescents with ASD completed the intervention. There was a statistically significant increase in steps of dental examination completed for children (5.4) and adolescents (5.2). Regarding behaviour, the median in the pretest was 2 (negative behaviour) for children and adolescents, and in the posttest, this increased to 3 (positive behaviour) for children and 4 (very positive behaviour) for adolescents; this difference was statistically significant. The maintenance test 1 month after the posttest showed that the improvement in the number of steps was maintained.	40
			Before and after					

Parry et al. (2018)	To involve all key stakeholders to gain an understanding of oral health challenges associated with delivering and receiving oral health care, with a view to aiding improved dental experience and reattendance in the primary care dental setting for patients with ASC.	UK, Sussex	Qualitative descriptive		Special school, local dentists; interviews, focus group.	Parents (*n* = 7), children (*n* = 3), dentists (*n* = 5) and dental nurses (*n* = 3). Age (children of parents): 5–14 years.	ACCESS: Sensory difficulties, dentist expectations and attitudes and lack of previsit preparation for dental visits were highlighted as important factors influencing dental experiences for children with ASC and their families. Preferable to reduce the time spent in the waiting room, for example, be given the first appointment.	100
			Qualitative					
							PROVISION: Dentists described how efforts to meet NHS targets can restrict the likelihood of longer or more frequent familiarization appointments being booked. They may be unaware of the child's ASD diagnosis (parents commented that not asked to report diagnosis on medical form). It is worthwhile to invest time and to build rapport with patient—pays off with more acceptance of treatment.	
Popple et al. (2016)	To assess the effectiveness and feasibility of a digital dental education programme for autistic children and caregivers; to gauge parent interest in incorporating the programme into the child's home routine.	USA, New Haven	Randomized crossover trial—pilot RCT	Use of video to model proper brushing with narration and closed captioning. Email reminder sent to participants twice a day for 3 weeks. Three visits to dentist to evaluate oral hygiene.	Paediatric Dental Clinic; questionnaire, dental exam.	Children (*n* = 18—9 cases, 9 controls). Age: Mean case 8.78, control 8.89. Gender: Male—case 66.6%, control 44.4% Social Responsiveness Scale—2: case 82.11, control 73.0	ORAL HEALTH: Children's oral hygiene improved in both groups, with ‘suggested’ greater improvements in the treatment group.	20
Stein Duker et al. (2019)	To qualitatively explore parent and dentist reports of successful strategies implemented during dental care with children with ASD.	USA, Southern California	Qualitative descriptive		Local schools and hospitals; focus groups.	Parents (*n* = 9. Dentists (*n* = 7). PARENTS maternal education level: High school or GED 33.3%, college 22.2%, Graduate degree or above 44.4%. Paternal education level: High school or GED 33.3%, college 33.3%, Graduate degree or above 33.3%. DENTISTS: Type of dentist: General 14.3%, Paediatric 85.7%. How frequently treat children with ASD: Occasionally 14.3%, Often 28.6%, Very often 57.1%. Treated how many children with ASD in the last 2 years of practice: 10 14.3%, 50–99 28.6%, 100 or more 57.1%.	ACCESS: Importance of having a ‘good dentist’—calm, understanding, supportive regardless of the behaviour of the child. Experience of autism. Positive re‐enforcement and attitude of the dentist, flexibility, strategic scheduling of visits, co‐ordination with other health professionals, individualization of care and appropriate preparation.	100
			Qualitative					
							PROVISION: Parent knows best—utilization of parents' knowledge of the child, its behaviours and triggers and motivators. Use of familiarization and desensitization to diminish aversion to the dental clinic. Being flexible to accommodate the needs of the children with ASD—taking an individualized approach. Networking and learning from other colleagues.	
Stein Duker et al. (2017)	To provide an increased understanding of the challenges experienced during oral care in the dental office by children with ASD.	USA, Southern California	Qualitative descriptive		Local schools and hospitals; focus groups.	Parents (*n* = 9).	ACCESS: Difficult to find the right dentist (dentist rejection, dentist misrepresentation, challenge of obtaining referrals, cost). Sensory sensitivities. Use of restraint. Use of drugs.	100
						Maternal education Level: High school or GED 33.3%, college 22.2%, graduate degree or above 44.4%. Paternal education Level: High school or GED 33.3%, college 33.3%, graduate degree or above 33.3%.		
			Qualitative					
Stein et al. (2014)	To investigate the behavioural and physiological stress and anxiety in children with ASD during routine oral care and determine if there are factors other than an ASD diagnosis that are associated with behavioural and physiological distress.	USA, Southern California	Nonrandomized study		Children's Hospital dental clinic; parent and dentist reports, video, physiological testing.	CHILDREN (*n* = 44—22 ASD; 22 TD). TD/ASD. Age: 6–12, mean: 8.3/8.2. Gender: Male 45.5%/81.8%. Race: Caucasian 95.5%/81.8%, not Caucasian 18.2%/4.5%. Ethnicity: Not Hispanic/Latino 31.8%/18.2%, Hispanic/Latino 68.25/81.8% Maternal education level: High school, GED, or less 40.9%/18.2%, Vocational/associates/college courses 27.3%/68.2%, Bachelor's degree or more 31.8%/13.6%. Paternal education level: High school, GED, or less 7 33.3%/52.4%, Vocational/associates/college courses 33.3%/23.8%, Bachelor's degree or more 33.3%/23.8%.	ACCESS: ASD children show significantly more uncooperative behaviours during routine dental cleanings and significantly higher electrodermal arousal compared to TD children, indicating greater physiological stress during dental cleaning. In ASD children, lower expressive communication ability and physiological distress are correlated with uncooperative behaviour.	60
			Case‐control					
Stein et al. (2013)	To investigate the relationship between sensory sensitivities and oral care difficulties in autistic children or TD and to compare autistic children categorized as ‘Sensory Over‐Responders’ (SOR) with autistic children not ‘Sensory Over‐Respondent’ (SNOR).	USA, Southern California	Nonrandomized study, case‐control		Schools, questionnaire	Parents of TD (*n* = 202) and ASD (*n* = 196) children.	ORAL HEALTH: 65% of parents of children in the SOR group reported difficulty with their child's oral care (toothbrushing) daily versus 47% in the SNOR group (*p *= .04). Significantly more people in the SOR group reported that their child disliked the taste/texture of toothpaste in their mouth and the child showed gagging during toothbrushing (*p* = .002, *p *= .006, respectively); No significant difference between SOR and SNOR children disliking the feeling of a toothbrush in the mouth (*p *= .15).	20
							ACCESS: Significantly more people in the SOR group reported that it was moderately to extremely difficult to have the dentist or hygienist clean their child's teeth vs the SNOR group (65% vs. 39%, *p*= .004). More people in the SOR group reported that their child's last experience at the dentist was negative (50% vs. 31%, *p*= .03). 41% in the SOR group reported that if their child had to go to the dentist tomorrow for dental prophylaxis, he/she would be afraid or extremely afraid, versus 26% of the SNOR group (*p *= .08). Significantly more people in the SOR group reported their child's sensory sensitivities increased in the dental office (56% vs. 26%, *p* = .001) and that those sensory sensitivities made dental appointments challenging (73% vs. 42%, *p* = .0005).	
Stein et al. (2012)	To examine sensory‐related aspects of oral care at home and the dentist's office in autistic children.	USA, Southern California	Mixed methods		Children's hospital and schools; questionnaire.	Parents of TD (*n* = 202) and ASD (*n* = 196) children (survey). Mothers of children with ASD (*n* = 5) (focus group).	ORAL HEALTH: (TD/ASD % Odds ratio, **p*< .05, ***p* < .0001)—Difficulty with toothbrushing in the home: 10/61** 17.8, Dislike of taste or texture of toothpaste: 20/55* 9.5, Dislike of feeling of toothbrush in the mouth: 25/57* 8.1	60
			Cross‐sectional and qualitative					
							ACCESS: (TD/ASD % Odds ratio, **p* < .05, ***p* < .0001). Parent reported ‘moderate to extreme’ difficulty with cleaning at dental office 13/60** 15.0. Sensory sensitivities increased at the dental office. 6 /47** 16.3. Uncooperative behaviours increased at the dental office 4/49** 31.1. Sensory sensitivities made dental appointments challenging ASD only 46%. Uncooperative behaviours made dental appointments challenging ASD only 45%.	
Stein et al. (2012)	To investigate the differences between children with ASDs and their typically developing peers (TD) in relation to aspects of oral care.	USA, Southern California	Nonrandomized study		Children's hospital and schools; questionnaire.	Parents of TD (*n* = 202) and ASD (*n* = 196) children (TD/ASD%) maternal education level: High school or GED 16/18, college 41/46, graduate degree or above 41/32, not reported 2/4. Paternal education level: High school or GED 25/21, college 35/45, graduate degree%2B 34/23, not reported 6/11.	ORAL HEALTH: 61% of ASD children reported difficulty with their child's toothbrushing versus 10% TD (*p *< .001). ASD children brushed their teeth significantly fewer times per week than TD children (mean = 10.5 ± 4.8, 12.6 ± 3.9, respectively; *t* (*df* = 372) = −4.56, *p* < .001). More than half of the ASD children required ‘some or complete’ physical assistance with toothbrushing versus 28% TD children.	80
			Cross‐sectional					
							ACCESS: Significantly more parents of ASD children rated their child's last experience at the dentist's office as ‘negative’ (38% vs. 4%; *p* < .001) than TD children and that it was ‘moderately to extremely’ difficult to have the dentist or hygienist clean the child's teeth (60% vs. 13%; *p* < .001). Significantly more ASD children than TD children were afraid of, disliked or complained about dentist drilling; bright lights; loud sounds; having someone put instruments in his or her mouth; leaning back in the dentist's chair; and smells (*p *< .001 for all variables). Significantly more ASD children (52%) reported difficulty with three or more of these sensory variables versus 6% TD children (*p *< .001).	
Stein et al. (2011)	To examine oral care difficulties and sensory sensitivities in children with ASD and other disabilities.	USA, Southern California	Quantitative descriptive		Parents who received services from the Paediatric Therapy Network (PTN); questionnaire.	Parents of children receiving services from PTN (*n* = 206).	ORAL HEALTH: 41.9%, 48.0% and 39% reported that children with ASD Only, ASD Plus and Other Disability, respectively, had difficulty with oral care at home (not statistically significantly). A statistically significant between‐group difference was found for dislike of the feeling of the toothbrush in the child's mouth when comparing the ASD Plus group and the other Disability group. Within the ASD Plus group, dislike of taste of toothpaste, dislike of feeling of toothbrush in the mouth, sensory sensitivities in dental office each statistically significantly associated with difficulty with home‐based care.	20
			Cross‐sectional					
							ACCESS: Difficulty children experienced getting their teeth cleaned in the dental office—no significant difference between groups. Statistically significant difference between ASD Only (48.3%), ASD Plus (44.4%) and Other Disability (28.9%) regarding behaviour difficulties. Statistically significant difference between ASD Only (63.0%) and ASD Plus (49.2%) versus Other Disability group (30.8%). Within the ASD Plus group, dislike of the feeling of the toothbrush and sensory sensitivities in the dental office were statistically significantly associated with difficulty with teeth cleaning in the dental office.	
Taghizadeh et al. (2018)	To explore the experiences of children with ASD who underwent a day surgery procedure.	Australia, Melbourne	Mixed methods		Hospital Dental Clinic; interviews, review patient records.	Caregivers (*n* = 15) of children with ASD scheduled for an elective day surgery procedure; health care providers (*n* = 14) involved in the care of the children interviewed. Health care provider roles: admission clerk, admission nurses, anaesthesiology nurses and technicians, anesthesiologists, anaesthesiology resident, post anaesthesia care unit nurses and play therapist.	ACCESS: Barriers—prolonged waiting times for operation date and waiting on day of operation, lack of private space, lack of noninvasive equipment, poor communication and inadequate training of staff about ASD. Facilitators—good communication, clear explanations and friendly attitudes of staff. Flexibility and individualized care of patients valued. Supportive aids, use of social stories and premedication helpful.	100
			Qualitative descriptive/cross‐sectional					
Taneja et al. (2020)	To investigate perceived barriers to accessing dental care that are specific to caregivers of children with ASD.	USA, Connecticut	Nonrandomized study		Hospital for Special Care; questionnaire.	Caregivers of children with ASD—cases (*N* = 46), Caregivers of children without ASD, but with chronic health issues—controls (*N* = 37). Men and women aged 40–65 years. 56.5% of cases and 63.5% of controls attended at least high school.	ORAL HEALTH: (%) Cooperative at home for routine brushing. Cases: 87.0/controls: 65.2.	40
			Cross‐sectional				ACCESS: Mean number of reported barriers to care significantly higher in cases than controls (2.10 vs. 0.76. *p*= .00025). Significantly higher number of cases reported that difficulty with cooperation was a barrier to finding dental care vs controls (*p *= .0007). Finding a dentist comfortable treating their child significantly higher rate in cases than controls (*p *= .019). 28.3% of cases reported it hard to find a dentist who takes their dental insurance versus 5.4% of controls. 40% of cases reported their child's behaviour hindered care. (%) Cooperative behaviour at the dentist's clinic: cases 73.9%/controls 86.5%.	
Thomas et al. (2018)	To gather dental experiences of UK parents of children with autism or working diagnosis of autism and explore how they feel primary care dental services can be improved.	UK, Somerset/Devon	Qualitative descriptive		Local autism support groups, patient and public involvement group; interviews.	Parent carers (*n* = 16). Female 100%. Ages 20–50%2B years.	ACCESS: Good communication; clear pathway to specialized dental service; parent/carer confidence; continuation of service; dental team flexibility.	100
			Qualitative					
Weil et al. (2011)	To explore (a) the attitudes and behaviours of members of the Special Care Dentistry Association (SCDA) who self‐identified as treating patients with ASD and (b) the relationship between their professional attitudes and behaviours concerning these patients.	USA, National	Quantitative descriptive		SCDA; on‐line survey.	SCDA members who treat patients with ASD (*N* = 75). Age: 28–85 years, mean age: 49 years. Gender: male 49%. Ethnicity: 84% European American,10% Asian American, 4% African American. Dental degree 83%. Speciality education 35%. Mean length of time of practice 23 years. Practice location: 10% rural/small town, moderate city 31%, Suburb 20%, Large city 39%. Percentage of patient population high SES 18%, average 31%, Low SES 53%. Percentage of patients covered by Medicaid 48%, Private pay 21% and Insurance 25%.	PROVISION: 47.7% disagree/strongly disagree that ‘my professional education prepared me well for treating patients with ASD’. 57% agree/strongly agree that ‘I like to treat children with ASD’. 72.3% agree/strongly agree that ‘My dental team members are comfortable treating patients with ASD’. 58.4% agree/strongly agree that ‘My dental team members are knowledgeable about treating patients with ASD’. 35.4% agree/strongly agree that ‘Patients with ASD are often unable to tolerate dental treatment’. 72.3% agree/strongly agree that ‘Financial compensation for treating autistic patients is inadequate’. Special accommodations made for ASD patients: 59% use special visual aids for communication and 90% use rewards at the end of the child's visit. 78% give special instructions to parents before treatment. 91% allow parents to accompany child in the operatory. 71% allow familiarization visits before the first appointment. 80% have special scheduling arrangements.	60
			Cross‐sectional					
Weil et al. (2010)	To explore general and paediatric dentists' professional attitudes and behaviours towards patients with ASD; their perceptions of their dental education about these issues; and the relationships among their educational experiences, attitudes and behaviours concerning patients with ASD.	USA, National	Quantitative descriptive		Members of the Michigan Dental Association and the American Academy of Paediatric Dentistry; on‐line survey.	Paediatric dentists (*n* = 212) and general dentists (*n* = 162).	PROVISION: The respondents disagreed with statements indicating that their predoctoral dental education had prepared them well to treat patients with ASD. The more respondents felt prepared, the more likely they were to provide care for ASD patients. The frequency with which paediatric dentists said they use appropriate behaviour management strategies when treating patients with ASD correlated with the quality of their educational experiences.	60
			Cross‐sectional					
Yost et al. (2019)	Follow‐up study (see Nelson) to examine: (1) ability to accept an examination after 2 years following initial success; (2) quantify new dental skills acquired; and (3) use of advanced behaviour guidance techniques during the study period.	USA, Seattle	Nonrandomized study Retrospective case series	Desensitization programme with individualized care plan that included goal setting for future visits and previsit home preparation. At each visit, behaviour guidance techniques were used.	Hospital paediatric dental clinic; chart abstraction.	Children with ASD who participated in dental desensitization programme. *N* = 138, Age: 4–18 years. Gender: Male 83%. Race: Caucasian 50%, Asian 11%, African American 9%, Other/multiple 17%, Unanswered 12%. Insurance: Public 55%, private 43%, none 1%.	PROVISION: Strategies when treating patients with ASD correlated with the quality of their educational experiences. No behavioural variable was significantly associated with maintaining the ability to receive an MTE during the study period. No significant associations between patient characteristics and ability to the main MTE during the study period.	40
Zhou et al. (2020)	Impact of social stories used to teach toothbrushing skills for preschool children with special needs on the toothbrushing performance and oral hygiene status of autistic and nonautistic children.	Hong Kong	Nonrandomized study	Validated toothbrushing social story used to demonstrate the toothbrushing procedure to children. After clinical examination and toothbrushing assessment, a social story‐assisted toothbrushing training was provided to all participants. Parents/carers were encouraged to read social stories to their children before or during daily toothbrushing once per day for 6 months.	Special Child Care Centre; dental exam, toothbrushing assessment, questionnaire.	Parent/children—children with autism (*n* = 87) children with special care need without autism (*n* = 94). Age: Mean (years) 3.79 ± 0.84. Gender: Male 69.6% Intellectual impairment: Unspecific: 27.1%, mild: 43.1%, moderate to severe: 29.8%. Levels of practical skill: Average or high: 6.6%, limit: 53.0%, low: 40.3%.	ORAL HEALTH: At baseline, for both groups, four toothbrushing steps were performed and less than 99 s spent in a single toothbrushing session. After the intervention, six steps were performed by children without additional assistance. Toothbrushing performance, oral hygiene and gingival status of the children were significantly improved after using social stories. Autistic children had better oral hygiene status (*p* = .01) and better gingival status (*p* < .001) than nonautistic children. No significant differences in toothbrushing performance among autistic and nonautistic children. Regression models indicated that improvements of children's toothbrushing performance and oral health status were associated with children's intellectual functioning and parents' attitudes towards the usefulness of social story intervention.	80
			Before and after study					

Rohani et al. (2018)	To assess the oral health behaviours of children with ASD and explore attitudes and barriers in providing oral care by their parents.	Malaysia, Kuala Lumpur	Mixed methods		University Paediatric Dental Clinic; questionnaire, interview.	Parents (*n* = 20). Average monthly income: RM 2000‐RM 5000 35%, RM 5000‐RM 10,000 50%, More than RM 10,000 15%.	ACCESS: Factors that affect anxiety or decrease co‐operation in child with ASD during the dental visit (could give more than one answer): Noisy equipment 50%, unfamiliar sensation 50%, bright light 15%, child dislikes physical contact 10%, challenging behaviour 10%.	20
			Cross‐sectional					
			Qualitative					
Fageeh et al. (2021)	To assess the effectiveness of ABA to improve knowledge regarding oral hygiene practices among cooperative autistic children.	Saudi Arabia, Jazan	Nonrandomized study	Mobile app programmed with videos on oral hygiene.	Special care school; questionnaire.	Children with ASD (*n* = 15) aged 6–12 years and their parents.	ORAL HEALTH: Significant increase in the mean oral hygiene knowledge score between baseline and 4 weeks—for children, increased from 4.73 to 9.00 (*p *< .001), and for parents, increased from 9.3 to 14.6 (*p *< .001).	
			Cross‐sectional					
Florindez et al. (2021a)	To explore what Latinx caregivers learned about their child's diet preferences and food routines in relation to their oral health. As a secondary aim, the study sought to explore whether notable differences in diet emerged between Latinx children with and without ASD.	USA, Los Angeles	Qualitative descriptive		Community; food journal, interview.	Participants were 32 Latinx caregivers from 18 families with children with and without Autism (*n* = 8 with a typically developing child and *n* = 10 with a child with ASD).	ORAL HEALTH: Visual methodologies highlighted the negative impact of consuming too much sugar on oral health.	
			Qualitative study with inclusive visual methodologies					
Florindez et al. (2021b)	To examine the knowledge, attitudes and practices of oral care of Latinx parents/caregivers of children with or without ASD to identify gaps to focus future intervention.	USA, Los Angeles	Quantitative descriptive		Community; questionnaire.	Parents/caregivers, Latinx with child 4–14 years old. (children TD [*n* = 29] children with ASD [*n* = 31]). 90% female, a mean age of 38.6 years (standard deviation [SD]: 6.5 years), 73% of parents, 95% of children had dental insurance. Mean age of children 8.5 years (SD: 3.2 years).	ORAL HEALTH: Mean knowledge scores and oral health habits not significantly different between ASD and TD children's parents.	
			Cross‐sectional					
							ACCESS: Fear of dentist significantly correlated with ASD. Difficulty in finding dentist significantly associated with ASD (*p*< .005). Latinx parents/caregivers of children with and without ASD report barriers to dental care, including difficulty attending visits or feeling stigmatized by their dental provider due to their ethnicity.	
Kind et al. (2021)	To assess if Dutch children with ASD regularly visit a dentist and to evaluate parents' satisfaction with the care provided.	The Netherlands, National	Quantitative descriptive		Regular practices, paediatric dentists and special needs dental centres; interviews.	Parents of ASD children aged 2–18 years (*n* = 227).	ACCESS: 15% of the children did not receive the needed care when they had toothache and 21% of parents were unsatisfied with the current dental care provided. No difference was found between satisfied and unsatisfied parents in the type of dental practice visited (*p *> .05). Children of unsatisfied parents reported pain more often during the last year (*p *= .013) and had a more severe type of ASD (*p *= .016).	
			Cross‐sectional					

McMillion et al. (2021)	To investigate the strategies that UK‐based specialist dental professionals use when working with autistic patients.	UK, National	Qualitative descriptive		Known dental professionals; interviews.	Dental health professionals (*n* = 16). Paediatric dentist: 31%, Orthodontist: 25%, Special care dentist: 18.8%, Dental therapist: 6.3%; Female: 75%.	PROVISION: Themes: Unique dental needs associated with being autistic; effective adaptations to practice; and crucial role of caregiver; importance of specialist knowledge. To improve dental care need to involve patients in decisions making about their treatment; flexibility; willingness to work with autistic patients and their caregivers.	
			Qualitative					
Parry et al. (2021)	To examine parental perceptions of difficulties associated with dental attendance and oral care for autistic children and young adults, to highlight reported challenges and potential adaptations, to identify interventions that will encourage positive experiences of dental attendance.	UK	Qualitative descriptive		Primary and secondary schools; focus groups.	Parents of primary and secondary school children with ASD (*n* = 6).	ORAL HEALTH: Importance of positive oral health messages; lack of understanding regarding the complexity of changing ritualistic dietary regimes and enacting good dental habits.	
			Qualitative					
							ACCESS: Need for understanding & training; awareness of sensory issues; recognition of individuality of autistic traits; time & clarity for communication; factors affecting the confidence of parents to advocate in the clinical environment	
Teste et al. (2021)	To evaluate the difficulties encountered by parents in maintaining oral hygiene in autistic children and the solutions that they found to facilitate this daily act.	France, National	Quantitative descriptive		Associations for autistic children; questionnaire.	Parents of children with ASD (*n* = 756)	ORAL HEALTH: Girls were 1.7 (95% CI: 1.1–2.8) times more likely to have toothbrushing difficulty than boys. Nonverbal children (OR: 3.2; 95% CI: 2.2–4.9), syndromic autistic children (OR: 2.8; 95% CI: 1.4–5.2), children using pictograms (OR: 1.6; 95% CI: 1.1–2.4) and younger children (OR: 0.9; 95% CI: 0.9–0.9) were significantly more likely to encounter difficulties in tolerating toothbrushing. Parents used planning, modelling and making it enjoyable to facilitate toothbrushing. 79% of parents did not feel sufficiently informed about the different oral hygiene prevention tools and techniques.	
			Cross‐sectional					
Zheng et al. (2021)	To evaluate the acceptability and effectiveness of Cheerbrush.	USA, Tennessee	Nonrandomized study	Interactive augmented reality coaching system, which allows children to manipulate virtual objects like a toothbrush and toothpaste with their hand motions to practice the steps of toothbrushing. Demonstrates steps using audio and visual cues, while showing the brushing process through a virtual avatar. Assesses toothbrushing skills with a mechatronic toothbrush.	Clinical registry, local community; observation, movement and physiological testing.	Children with ASD (*n* = 6) and children with TD (*n* = 6).	ORAL HEALTH: Improvements in toothbrushing motions and reduced stress for the children in the posttest. Posttest questionnaire showed that most children (ASD & TD) enjoyed interacting with CheerBrush. Parents (of AD & TD children) enjoyed CheerBrush and found the instructions beneficial for their children.	
			Case‐control					
Abomriga (2017)	To provide an in‐depth exploration of children's oral care experiences and concerns of parents of children living with ASD.	Canada, Montreal	Qualitative descriptive		‘Private centres that deal with children with ASD’; interviews.	Parents *N* = 6.	ORAL HEALTH: Three broad findings in the care of their child's oral health, namely, ‘Oral care as a struggle’, ‘struggling with(out) giving up’ and ‘Oral care as a Hope’	100
			Qualitative					

Kooijman (2016)	To explore the perceived barriers of special dental care providers in oral hygiene of all children with ASD.	The Netherlands	Qualitative descriptive		Members of specialist dental associations; questionnaire, interviews.	48 Oral health care providers for patients with special needs—15 paediatric dentists and 14 dentists specialized in dental care for disabled and 19 oral hygienists.	PROVISION: Main perceived barriers for practitioners in care for children with ASD were: Communication/Contact, Sensory perception and Cooperation, Knowledge about ASD. Perceived culture‐related barriers in home oral care were nutrition and sugar intake and support and attitude. Language was a barrier in the professional oral care in children with ASD from various cultural backgrounds.	100
			Cross‐sectional					
Sahab (2017)	To examine the factors that predict dental anxiety in children with ASD.	UK, Reading	Mixed methods		Centre for autism and local support groups; questionnaire, interviews.	Parents (*n* = 92), children (ASD = 45, TD = 47), dentists (*n* = 7). Children 11–17 years.	ACCESS: Dental anxiety is related to parental anxiety, sensory sensitivity, worries about pain and negative experiences.	100
			Qualitative descriptive and nonrandomized study					
